# Urinary Biomarkers in Urological Oncology: From Biological Origin to Clinical Decision-Making—A Narrative Review

**DOI:** 10.3390/diagnostics16172810

**Published:** 2026-09-01

**Authors:** Aliya Nurmukhanbetova, Kassymkhan Sultanbekov, Khussan Umurzakov, Zie Gassanov, Altyn Duisenbayeva, Alimkhan Talas, Lazzat Shaikenova, Aidos Bolatov

**Affiliations:** 1 General Immunology Department, Asfendiyarov Kazakh National Medical University, Almaty 050054, Kazakhstan; nurmukhanbetova.a@kaznmu.kz; 2 Department of Public Health and Healthcare, South Kazakhstan Medical Academy, Shymkent 160019, Kazakhstan; 3 Urooncology Center, Kazakh Institute of Oncology and Radiology, Almaty 050022, Kazakhstan; ziegasanov@mail.ru; 4 Department of General Medical Practice No. 2, Asfendiyarov Kazakh National Medical University, Almaty 050054, Kazakhstan; d.altyn_zh@mail.ru (A.D.); albergerectino@gmail.com (A.T.); 5 Department of Clinical Disciplines, University of International Business Named After Kenzhegali Sagadiev, Almaty 050010, Kazakhstan; lazzat.shaikenova1965@gmail.com; 6 Shenzhen University Medical School, Shenzhen University, Shenzhen 518037, China; bolatovaidos@gmail.com; 7 Department of Science, “University Medical Center” Corporate Fund, Astana 010000, Kazakhstan

**Keywords:** urinary biomarkers, liquid biopsy, bladder cancer, upper-tract urothelial carcinoma, prostate cancer, renal cell carcinoma, biomarker validation, precision oncology

## Abstract

Urinary biomarkers are promising tools in urological oncology because urine can be collected non-invasively and repeatedly during diagnosis, treatment, and surveillance. Despite extensive biomarker discovery, their adoption in routine practice remains limited. Biomarker performance depends not only on analytical accuracy but also on the clinical decision, anatomical route into urine, biological source of the signal, specimen fraction, and timing and conditions of sampling. This review evaluates urinary biomarkers across bladder cancer, upper-tract urothelial carcinoma, prostate cancer, and renal cell carcinoma using a biologically grounded, decision-oriented framework. It considers tumor-derived nucleic acids, epigenetic alterations, proteins, immune and inflammatory mediators, extracellular vesicles, metabolites, microbiome-associated signals, and multiparametric models. Bladder cancer represents the most anatomically direct and clinically mature setting, with potential applications in hematuria evaluation, cystoscopy triage, recurrence surveillance, molecular residual disease assessment, and monitoring of response to bacillus Calmette–Guérin therapy. In upper-tract urothelial carcinoma, distinguishing voided from selectively collected urine is essential because dilution, transit, obstruction, and limited localization affect interpretation. Prostate urine assays are best positioned for biopsy triage and refinement of active surveillance rather than general population screening. Renal cell carcinoma biomarkers remain exploratory because the sources and mechanisms of urinary signal release are insufficiently resolved. Clinical translation should be assessed using decision-specific outcomes, including incremental value, calibration, net clinical benefit, procedures avoided, significant cancers missed, reproducibility, and feasibility, rather than diagnostic accuracy or area under the receiver operating characteristic curve alone.

## 1. Introduction: Why Urinary Biomarkers Remain Difficult to Translate into Clinical Oncology

Urine is an attractive liquid-biopsy medium in precision oncology because it can be collected non-invasively, repeatedly, and at virtually every stage of cancer care, from initial diagnosis to treatment monitoring and long-term surveillance [1,2]. Unlike tissue biopsy, urine sampling imposes minimal patient burden and readily supports longitudinal assessment. In urological malignancies, urine is particularly appealing because of its anatomical relationship to the urinary tract and because it contains diverse classes of biomarkers, including exfoliated cells, DNA, RNA, extracellular vesicles (EVs), proteins, metabolites, microbial products, and conventional cytology [3,4].

Despite these advantages, translation into routine clinical oncology has been considerably slower than anticipated. Numerous urinary assays have demonstrated promising analytical performance, and several have received regulatory approval, yet few have substantially altered clinical management. In bladder cancer, cystoscopy remains the cornerstone of diagnosis and surveillance despite decades of urinary biomarker research [5,6]. Clinical implementation is even more limited in upper-tract urothelial carcinoma (UTUC), prostate cancer, and renal cell carcinoma (RCC), where urinary biomarkers remain largely adjunctive or investigational [7,8].

This translational gap is usually attributed to heterogeneous study populations, inconsistent specimen collection and processing, insufficient external validation, and poorly defined clinical use cases [1,3]. These limitations are important but do not fully explain why biomarkers with apparently similar analytical performance often show markedly different clinical utility across diseases and clinical settings. Several recent reviews have comprehensively summarized urinary biomarkers according to cancer type, molecular analyte, analytical platform, diagnostic performance, or stage of clinical development [3,4,5,6,7,8,9,10]. These approaches provide important descriptions of the available technologies and evidence base but do not fully address a complementary translational question: How should a urinary biomarker signal be interpreted before it can support a specific clinical decision?

Clinical interpretation is complicated because urinary signals may originate from multiple biological sources. Tumor-associated molecular alterations detected in urine do not necessarily derive from viable malignant cells. They may also reflect field-altered urothelium, treatment-related tissue injury, inflammation, renal pathology, benign epithelial turnover, or other non-malignant biological processes [11,12,13,14,15,16]. Consequently, identical analytical results may have different clinical implications depending on disease location, specimen collection, treatment status, and intended clinical use.

For clinical oncology, the central question is therefore not simply whether a urinary biomarker detects a molecular alteration, but whether that result can reliably support a specific clinical decision. The evidentiary requirements differ substantially between hematuria evaluation, cystoscopy deferral, localization of upper-tract disease, prostate biopsy triage, active-surveillance monitoring, molecular residual disease assessment, and treatment-response evaluation. A biomarker that performs well for one indication should not automatically be assumed to be appropriate for another.

**Figure 1:**
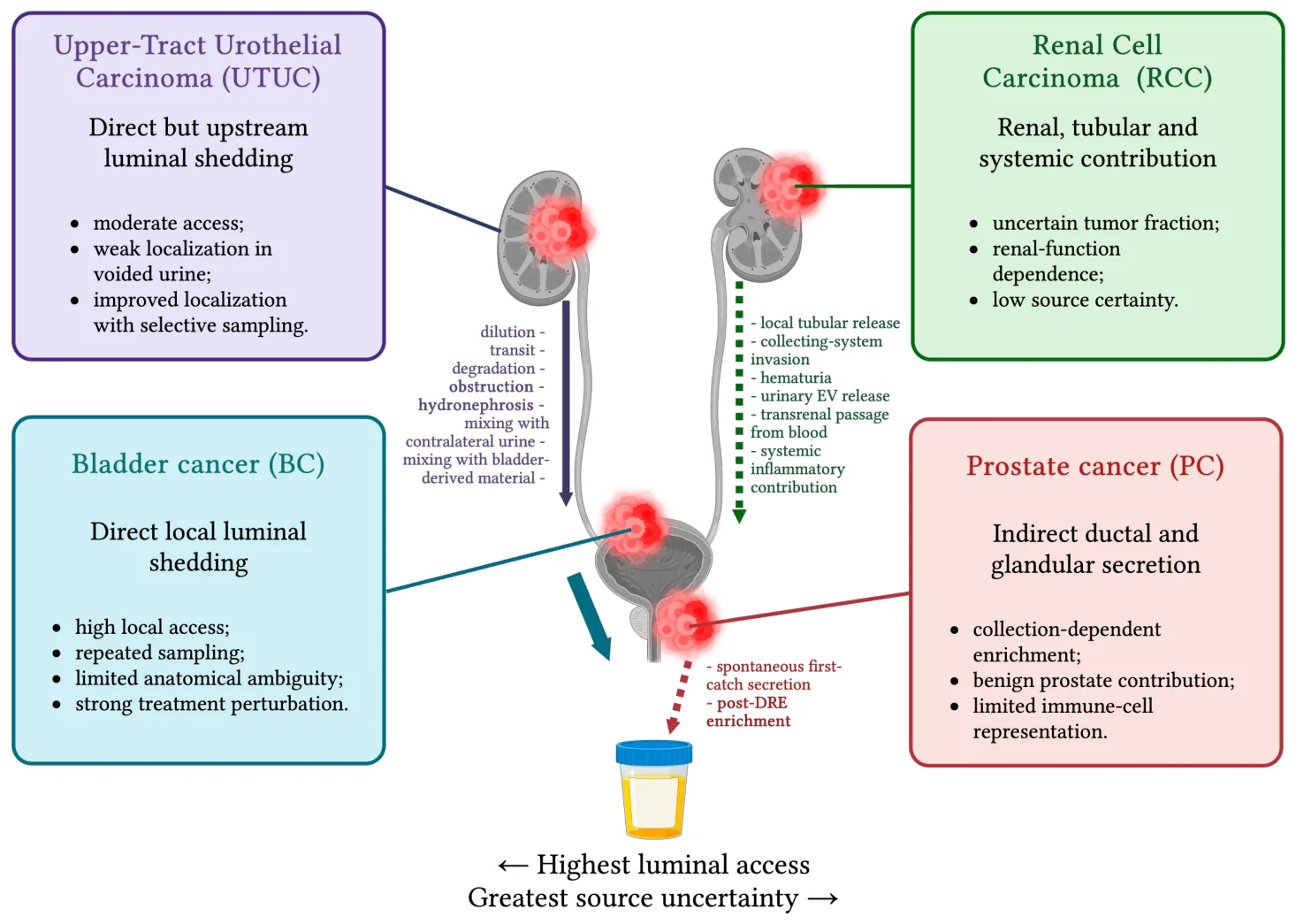
Anatomical routes by which cancer-associated signals enter urine across urological cancers. Bladder cancer has direct local luminal contact with urine; upper-tract urothelial carcinoma releases material upstream, where signals may be affected by transit and dilution; prostate cancer contributes material mainly through ductal secretion into first-catch or post-manipulation urine; and renal cell carcinoma may contribute urinary signals through renal parenchymal injury, collecting-system communication, hematuria, tubular handling, or systemic circulation. Solid arrows indicate direct luminal routes of urinary signal entry, whereas dashed arrows indicate indirect, collection-dependent, renal, or systemic routes. Connector lines link each cancer type to its anatomical site and do not represent routes of biomarker shedding. Arrow width is schematic and does not represent the magnitude of biomarker shedding or clinical performance. Abbreviations: RCC, renal cell carcinoma; UTUC, upper-tract urothelial carcinoma. Created in BioRender. Asanova, A. (2026) https://BioRender.com/anyp2hd (accessed on 26 June 2026).

The principal contribution of this review is therefore a biology- and clinical-decision-oriented framework that integrates five interrelated dimensions: anatomical access to urine, biological source, specimen fraction, perturbation and temporal context, and the clinical decision being addressed. Anatomical access differs substantially across urological cancers: bladder cancer is characterized by direct luminal shedding, UTUC by upstream luminal shedding, prostate cancer by ductal or manipulation-dependent secretion, and RCC by renal or systemic contribution to urinary signals (Figure 1) [17,18,19,20]. By applying this framework comparatively across bladder cancer, UTUC, prostate cancer, and RCC, we seek to distinguish analytical detectability from biological interpretability and clinical utility. The framework is further extended to biomarker discordance and to practical evidence requirements for clinical translation. Accordingly, rather than providing another catalogue of urinary biomarker classes, this review provides a structured approach for determining not only whether a urinary signal is detectable, but what it represents, when it is informative, and which clinical decisions it can reasonably support.

### Review Methodology and Evidence Framing

This review was designed as a narrative evidence synthesis with a biological, translational, and clinical-decision focus, rather than as a systematic review or meta-analysis. The objective was not to compare the diagnostic accuracy of individual urinary biomarkers, but to develop a clinically oriented framework for interpreting urinary biomarker signals across bladder cancer, upper tract urothelial carcinoma (UTUC), prostate cancer, and renal cell carcinoma (RCC).

A structured literature search was conducted in PubMed/MEDLINE, Scopus, and Web of Science from database inception to 15 August 2026. In PubMed/MEDLINE, Medical Subject Headings (MeSH) and free-text terms were combined. Core MeSH terms included “Urine,” “Biomarkers, Tumor,” “Liquid Biopsy,” “Urinary Bladder Neoplasms,” “Carcinoma, Transitional Cell,” “Prostatic Neoplasms,” “Carcinoma, Renal Cell,” “Extracellular Vesicles,” “Cell-Free Nucleic Acids,” “DNA Methylation,” “MicroRNAs,” and “Metabolomics.” These were supplemented by disease-specific and biomarker-related free-text terms, including “bladder cancer,” “urothelial carcinoma,” “upper tract urothelial carcinoma,” “prostate cancer,” “renal cell carcinoma,” “urinary biomarker,” “tumor DNA,” “cell-free DNA,” “methylation,” “RNA,” “microRNA,” “protein,” “cytokine,” “extracellular vesicle,” “metabolomics,” and “microbiome.” Additional targeted searches addressed longitudinal monitoring, molecular residual disease, treatment response, clinical implementation, assay standardization, cost-effectiveness, artificial intelligence, and multimodal prediction. Equivalent keyword combinations were adapted for Scopus and Web of Science. Reference lists of relevant reviews and primary studies were also examined to identify additional eligible publications.

Eligible publications included peer-reviewed original clinical studies, prospective and retrospective biomarker studies, translational investigations, diagnostic and prognostic studies, systematic reviews, meta-analyses, consensus statements, and clinical practice guidelines relevant to urinary biomarkers in bladder cancer, UTUC, prostate cancer, or RCC. Studies were included when they provided evidence relevant to at least one component of the review framework: biomarker biological origin, anatomical accessibility to urine, specimen characteristics, analytical performance, clinical validity, longitudinal behavior, treatment response, or clinical implementation. Priority was given to studies with clinically characterized cohorts, clearly defined urinary specimens and analytical methods, and direct relevance to clinically meaningful applications, including hematuria evaluation, cystoscopy triage, recurrence surveillance, molecular residual disease assessment, treatment monitoring, UTUC localization, prostate biopsy triage, active surveillance, and renal-mass characterization. Studies were excluded if they (i) did not address urological malignancies; (ii) did not provide urine-based biomarker data relevant to the scope of the review; (iii) lacked sufficient methodological or clinical information for interpretation; (iv) were available only as abstracts or conference proceedings, or represented editorials or non-peer-reviewed reports; or (v) constituted duplicate reports without additional relevant data. Selected non-urine studies were considered when they provided directly relevant evidence on biological mechanisms, tumor evolution, complementary liquid-biopsy approaches, prediction methodology, or translational standards necessary to contextualize urinary biomarkers.

The literature identification and selection were conducted by the authors using the predefined scope and eligibility criteria described above. Because this review was designed as a narrative evidence synthesis rather than a systematic review, records were not subjected to formal independent duplicate screening or third-reviewer adjudication. Potentially relevant or uncertain publications were discussed among the authors, and inclusion was determined by consensus based on their relevance to the biological, clinical, and translational framework of the review.

Rather than organizing the evidence primarily by biomarker class or analytical platform, studies were interpreted using the biology- and decision-oriented framework developed in Section 2 and applied comparatively across the four urological cancers. Study selection and interpretation therefore emphasized biological source, anatomical accessibility to urine, specimen compartment, clinical context, and the decision that a biomarker is intended to inform. Because the purpose was conceptual and translational synthesis rather than exhaustive evidence enumeration or pooled effect estimation, no formal meta-analysis or systematic risk-of-bias assessment was performed; instead, the evidence was interpreted in relation to study design, validation status, clinical context, and translational maturity.

Throughout the review, evidence-supported clinical applications are distinguished from conceptual syntheses. Statements concerning established or emerging clinical applications are grounded in published clinical evidence and guideline-supported practice where available, whereas the proposed interpretive framework, evidence hierarchy, and recommendations for future study design and clinical translation represent the authors’ synthesis of the available literature.

## 2. A Decision-Oriented Compartment Framework for Urinary Biomarker Interpretation

Urine is often described as a single liquid-biopsy matrix, but in clinical practice it represents several biologically distinct sampling states. Voided urine collected during bladder-cancer surveillance, first-catch urine used for prostate biomarker testing, and renal pelvic urine obtained during ureteroscopy are all “urine” specimens, yet they differ in anatomical source, cellular composition, collection conditions, and clinical meaning.

In this review, we use a compartment-conditioned framework as an interpretive tool rather than as a formal evidence-derived classification. The framework considers five interacting dimensions: anatomical access to urine, biological source of the signal, specimen fraction, perturbation and sampling time, and intended clinical decision. These dimensions help clarify why the same analytical result may support different claims depending on whether the clinical task is hematuria evaluation, cystoscopy deferral, upper-tract localization, prostate biopsy triage, treatment monitoring, or molecular residual disease assessment.

### 2.1. Anatomical Access to Urine

The first determinant of urinary biomarker interpretation is the route through which tumor-associated material, host-response signals, or organ-derived injury markers enter the collected urine. Anatomical proximity alone does not determine clinical interpretability. Instead, each cancer type creates a different sampling condition, which affects what a positive or negative urinary result can reasonably support (Figure 1).

In bladder cancer, the tumor has direct luminal contact with stored vesical urine. This makes bladder cancer the most anatomically direct model for urinary biomarker development, because malignant cells, tumor DNA, methylated DNA, coding and noncoding RNAs, proteins, metabolites, EVs, cytokines, and recruited immune cells may be released directly into urine [21,22]. This direct access partly explains why urinary cytology, mutation assays, methylation tests, and transcriptomic panels are most developed in bladder cancer. However, direct contact does not equal tumor specificity: vesical urine also contains benign urothelial cells, leukocytes, erythrocytes, microbial products, renal-derived material, and proteins introduced through hematuria, infection, inflammation, or treatment-related injury [23].

In UTUC, tumor material also enters the urinary tract directly, but it originates upstream in the renal pelvis or ureter. Before reaching voided urine, the signal may be diluted, intermittently shed, retained by obstruction or hydronephrosis, or mixed with material from the bladder, urethra, contralateral kidney, and prostate. Voided urine therefore has limited ability to localize an upper-tract source. Selective renal pelvic or ureteral urine can enrich ipsilateral tumor-derived material and improve anatomical attribution, but it requires instrumentation and may introduce bleeding, epithelial injury, or saline dilution [17,24]. Thus, voided and selective urine are not interchangeable in UTUC, and urinary positivity must be interpreted alongside imaging, endoscopy, cytology, and the history of synchronous or previous bladder cancer.

In prostate cancer, most tumors do not communicate directly with the urinary lumen. Prostate-derived material enters urine mainly through prostatic ducts, glandular secretion, epithelial exfoliation, urethral mixing, and EV release. As a result, specimen collection becomes part of the biology of the test. DRE or prostate massage can enrich prostate-derived RNA, proteins, cells, and vesicles in first-catch urine, whereas some assays, such as ExoDx Prostate, use first-catch urine without mandatory DRE [14,18,19]. These collection states should not be considered equivalent. Prostate-enriched urine can support biopsy-triage decisions, but enrichment for prostate origin does not automatically establish tumor specificity because benign prostatic hyperplasia, prostate volume, prostatitis, and manipulation intensity may also shape the recovered signal.

RCC represents the least source-specific urinary biomarker setting. Candidate urinary signals may arise from tumor communication with the collecting system, hematuria-associated release, renal parenchymal injury, tubular secretion, EV transport, glomerular filtration of circulating molecules, systemic inflammation, or host-response biology [7,20]. Therefore, an RCC-associated urinary protein, RNA, DNA fragment, metabolite, or vesicle cannot be assumed to originate from malignant cells. Transrenal passage is particularly difficult to interpret because the mechanisms controlling filtration and urinary recovery of circulating DNA fragments and proteins remain incompletely defined [25].

These anatomical distinctions are intended as heuristic guides for clinical interpretation rather than quantitative rankings of biomarker performance and should not be interpreted as implying equivalent clinical maturity across cancers.

### 2.2. Biological Source of Urinary Signals

Analytical form should be distinguished from biological source. DNA, RNA, proteins, metabolites, and EVs describe what is measured or how a signal is packaged; they do not identify the cell or tissue from which the signal originated [11,26]. For clinical interpretation, this distinction is essential because a urinary alteration may be cancer-associated without being directly tumor-derived.

Five overlapping source categories are particularly relevant. First, malignant-cell-derived signals include somatic mutations, copy-number alterations, tumor-associated DNA methylation, fusion transcripts, dysregulated RNAs, tumor-associated proteins, intact malignant cells, and tumor-derived EV cargo [15,21]. The term tumor-derived should be used cautiously and, ideally, reserved for signals supported by tissue–urine concordance, tumor-informed mutation tracking, clonal phasing, or validated cell-of-origin analysis. Even then, detectability depends on tumor burden, anatomical access, shedding efficiency, treatment status, and specimen fraction [7,11].

Second, field-altered or injured epithelium can contribute cancer-associated signals without indicating viable malignant cells. In urothelial carcinoma, mutations or transcriptional alterations may extend into normal-appearing mucosa and persist after visible tumor removal [12]. Such field effects can complicate molecular residual disease assessment unless assays distinguish tumor-restricted alterations from alterations distributed across the urothelium [13]. Similarly, obstruction, instrumentation, surgery, intravesical therapy, renal tubular stress, and regenerative repair may release epithelial material that resembles tumor-associated signals.

Third, immune and inflammatory cells contribute cytokines, transcripts, proteins, metabolites, and EVs. Urinary immune-cell populations in bladder cancer may partly reflect the local tumor immune microenvironment, but similar signals can also arise from infection, catheterization, cystoscopy, ureteroscopy, or intravesical BCG [22,23,27]. Therefore, immune signals may provide prognostic, pharmacodynamic, or treatment-response information, but they should not be interpreted as direct evidence of malignant-cell release unless supported by additional tumor-specific markers or longitudinal clinical correlation [28,29].

Fourth, stromal and vascular compartments may contribute angiogenic proteins, extracellular-matrix fragments, cytokines, metabolites, and EVs associated with fibroblast activation, endothelial remodeling, and tissue repair. These signals may reflect tumor–host interaction, invasion, wound healing, obstruction, inflammation, vascular injury, or treatment-related repair. Urinary angiogenic and proliferation-associated protein profiles have been linked to urothelial field alterations and tissue remodeling, suggesting that some urinary signals represent the tissue ecosystem surrounding cancer rather than malignant epithelial cells alone [12].

Fifth, microbial, renal, and systemic context can substantially modify urinary composition. Microbiome-derived metabolites, inflammatory activation, renal filtration, tubular processing, hematuria, proteinuria, metabolism, and circulating host responses may all influence assay performance and biological interpretation [30]. These contextual signals may be clinically informative, but they should not be equated with malignant-cell presence without source attribution.

These source categories are not mutually exclusive. For example, an EV carrying a cancer-associated RNA transcript may originate from a malignant cell, field-altered urothelium, an immune cell, a stromal compartment, or renal tubular epithelium. Source attribution should therefore precede biological interpretation: an EV, RNA transcript, methylated DNA fragment, protein, or metabolite is not tumor-derived merely because it differs between cancer and control groups.

### 2.3. Specimen Fraction Determines the Sampled Biology

The term “urinary biomarker” is incomplete unless the analyzed specimen fraction is specified (Table 1). Fraction selection is not only a pre-analytical decision; it also determines which biological compartment is enriched and what clinical claim can reasonably be made from the result. Sediment enriches intact cells and cell-associated nucleic acids; supernatant enriches soluble and cell-free material; EV isolation enriches protected intercellular cargo; first-catch and post-DRE urine enrich prostate- and urethra-derived material; and selective upper-tract urine enriches ipsilateral renal pelvic or ureteral material. Therefore, a positive or negative result should always be interpreted in relation to the fraction analyzed.

Whole urine, sediment, supernatant, EV-enriched fractions, and selectively collected specimens interrogate different biological compartments. Sediment is suited to cytology and cell-associated mutation, methylation, and transcript assays, but depends on cell yield, centrifugation, processing delay, and storage conditions [15]. Supernatant-based assays are shaped by dilution, pH, osmolality, enzymatic activity, residual cellular contamination, and renal function [31]. EV-enriched fractions may preserve RNA, DNA, proteins, and lipids, but isolation methods recover different vesicle populations and remain difficult to compare across studies [32]. Selectively collected specimens, including first-catch, post-DRE, renal pelvic, ureteral, catheterized, and urinary-diversion urine, enrich specific anatomical sources but should not be considered interchangeable with standard voided urine. In UTUC, enrichment of exfoliated urinary cells may increase recovery of diagnostically informative material, but such approaches remain dependent on fraction choice and cell yield [33].

No single fraction should be assumed to represent the whole urinary compartment. Each fraction enriches different biological sources and creates different failure modes. For clinical oncology, the key question is therefore not which fraction is universally best, but which fraction is appropriate for a specified decision, such as hematuria evaluation, cystoscopy deferral, prostate biopsy triage, UTUC localization, or post-treatment monitoring.

### 2.4. Perturbation and Sampling Time

Urinary biomarkers are strongly influenced by when the specimen is collected in relation to disease activity, instrumentation, and treatment. For clinical oncology, sampling time is not a minor pre-analytical variable; it determines whether a urinary signal is more likely to represent baseline disease, procedure-related release, treatment-induced injury, pharmacodynamic response, or persistent malignant activity.

Two mechanisms are particularly important. First, procedures and treatments can generate urinary signal by releasing blood, injured epithelial cells, inflammatory mediators, immune cells, nonviable tumor DNA, and other tissue-derived material. Infection, hematuria, obstruction, hydronephrosis, and renal dysfunction may further modify urinary composition and analyte recovery [30,31]. In UTUC, obstruction and hydronephrosis may both reduce downstream passage of tumor-derived material and increase renal or urothelial injury signals [22]. Second, effective treatment may remove or suppress the biological source of a signal. Thus, an intervention can transiently increase tumor-associated material through mechanical disruption or cell death while reducing subsequent shedding by removing viable tumor.

This distinction is especially important after TURBT. Catheterization, cystoscopy, biopsy, ureteroscopy, and transurethral resection can cause epithelial disruption, bleeding, inflammation, and acute release of tissue-derived molecules [31,34]. Therefore, molecular positivity immediately after TURBT should not automatically be interpreted as residual disease. A persistent or re-emerging tumor-informed signal after an appropriate recovery interval is more clinically meaningful than isolated early post-procedural positivity [35,36].

During intravesical BCG therapy, urinary immune signals require similarly cautious interpretation. BCG intentionally induces local immune activation, including recruitment of myeloid and lymphoid cells and production of immune-associated transcripts and cytokines [37]. A strong urinary immune signal during treatment may therefore indicate pharmacodynamic engagement rather than tumor clearance. Conversely, a reduction in inflammatory signal may reflect resolution of treatment-induced cystitis rather than loss of antitumor activity. For BCG monitoring, urinary immune markers are most informative when interpreted longitudinally and, ideally, alongside tumor-informed molecular markers, cystoscopy, cytology, and clinical outcome.

Timing is also critical after kidney-sparing treatment for UTUC. Ureteroscopy, laser ablation, topical therapy, stent placement, and repeated instrumentation may generate hematuria, epithelial injury, and inflammatory signals while also changing the amount of tumor-derived material that reaches voided urine. In this setting, selective upper-tract and voided specimens should be interpreted as different temporal and anatomical sampling states, and persistent positivity should be distinguished from short-lived procedure-related release.

In prostate cancer, urinary biomarkers used during active surveillance should be interpreted in relation to prostate manipulation, biopsy timing, prostatitis, prostate volume, and MRI findings. A change in a prostate-enriched urinary RNA or EV signal may reflect grade progression, changing tumor volume, benign glandular contribution, inflammation, or sampling variability. Therefore, serial urinary testing in active surveillance should be evaluated as part of a decision pathway for repeat MRI, biopsy, or continued observation rather than as an isolated molecular trajectory.

For RCC, urinary signals after nephrectomy, ablation, or systemic therapy are particularly difficult to interpret because they may reflect loss of renal parenchyma, tubular injury, hematuria, systemic inflammation, treatment toxicity, or residual tumor biology [7,31]. Post-nephrectomy changes in urinary proteins, metabolites, EVs, or nucleic acids should therefore not be assumed to represent molecular residual disease without tumor-informed evidence, appropriate timing, and longitudinal association with recurrence or progression.

Overall, four temporal states should be distinguished: baseline samples collected before manipulation or treatment; acute perturbation samples collected shortly after TURBT, ureteroscopy, DRE, biopsy, BCG instillation, nephrectomy, ablation, or systemic therapy; recovery or washout samples collected after the immediate procedure- or treatment-related effects have subsided; and persistent or re-emerging signals after an initial decline. The last category is generally more compatible with residual or recurrent disease than isolated early positivity. Studies should therefore report the exact interval between sampling and recent instrumentation, surgery, prostate manipulation, intravesical therapy, systemic therapy, infection, hematuria, and renal-function change.

### 2.5. Intended Clinical Decision

**Figure 2:**
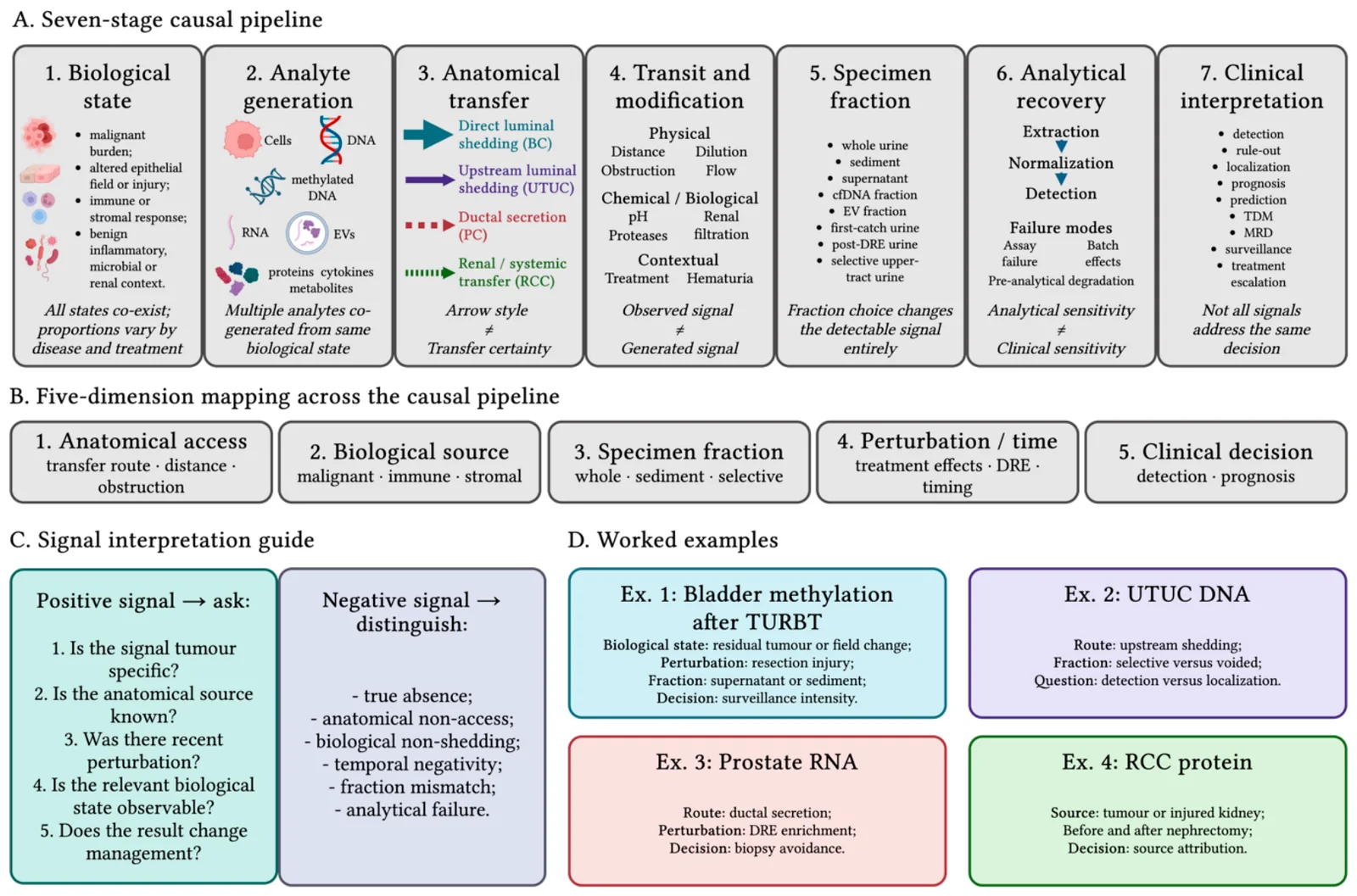
From biological state to clinical interpretation: a causal framework for urinary biomarkers. (**A**) The figure maps urinary biomarker interpretation from biological state to clinical decision, highlighting where anatomical transfer, transit, fraction choice, and assay recovery can alter meaning. (**B**) The five dimensions of the proposed interpretive framework are aligned with this causal pathway. (**C**) Positive and negative results require distinct interpretive checks, including source specificity, localization, perturbation, observability, fraction mismatch, and analytical failure. (**D**) Worked examples show how bladder methylation, UTUC DNA, prostate RNA, and RCC protein signals support different claims depending on biological and clinical context. Abbreviations: BC, bladder cancer; DRE, digital rectal examination; EV, extracellular vesicle; MRD, molecular residual disease; PC, prostate cancer; RCC, renal cell carcinoma; TDM, treatment-decision monitoring; TURBT, transurethral resection of bladder tumor; UTUC, upper-tract urothelial carcinoma. Created in BioRender. Asanova, A. (2026) https://BioRender.com/005f2a6 (accessed on 16 June 2026).

A urinary biomarker becomes clinically meaningful only when it is linked to a predefined oncological decision. The same analytical result may have different implications depending on whether the task is to detect cancer, defer an invasive procedure, localize disease, monitor recurrence, assess treatment activity, or support escalation. For this reason, urinary biomarker interpretation should move from the biological state to the clinical action through an explicit decision pathway (Figure 2).

Figure 2 summarizes how a urinary biomarker signal is transformed from an underlying biological state into a clinically interpretable result. The framework distinguishes seven sequential stages: the biological state generating the signal; generation of measurable analytes; anatomical transfer into urine; modification during transit; selection of the urinary specimen fraction; analytical recovery; and, finally, clinical interpretation (Figure 2A). Importantly, information can be altered or lost at several stages, such that the measured urinary signal is not a direct representation of the tumor itself. The five dimensions used throughout this review—anatomical access, biological source, specimen fraction, perturbation/time, and clinical decision—map onto this causal pathway and provide a structured approach to interpreting these sources of variation (Figure 2B). The framework therefore requires different reasoning for positive and negative results (Figure 2C): a positive result should be evaluated for tumor specificity, anatomical origin, recent perturbation, biological observability, and potential to alter management, whereas a negative result requires consideration of true absence as well as anatomical non-access, biological non-shedding, temporal variation, specimen-fraction mismatch, and analytical failure. The worked examples in Figure 2D illustrate why the same analytical result may support different clinical inferences across bladder cancer, UTUC, prostate cancer, and RCC. Thus, the framework is intended not as a biomarker classification system, but as a causal interpretive model linking biomarker biology and measurement to the specific clinical decision being addressed.

Diagnostic biomarkers identify currently existing disease in symptomatic or at-risk populations and must distinguish malignancy from infection, stones, inflammation, renal disease, and recent instrumentation [34,38]. Biopsy-triage biomarkers have a different role: they are not intended to replace histopathology, but to reduce unnecessary procedures while preserving sensitivity for clinically significant disease. In prostate cancer, tests such as ExoDx [39], SelectMDx [40,41], PCA3 [42], and the Michigan Prostate Score [43] are therefore best understood as decision-support tools for biopsy selection.

Endoscopy-triage and surveillance biomarkers should be judged by whether they can safely prioritize, defer, or intensify cystoscopy or ureteroscopy, particularly for high-grade urothelial carcinoma. Localization biomarkers are most relevant in UTUC and in patients with previous or synchronous bladder cancer, where urinary positivity may not identify the anatomical source of disease. Monitoring biomarkers track changes over time, whereas molecular residual disease assays seek persistent tumor-derived material after curative-intent treatment and require evidence of tumor origin, appropriate sampling time, and longitudinal association with recurrence [35,36].

Prognostic, predictive, and pharmacodynamic biomarkers should also be separated. Prognostic biomarkers estimate the future risk of recurrence, progression, or survival independent of treatment. Predictive biomarkers identify differential benefit from a defined therapy and therefore require treatment-specific validation. Pharmacodynamic biomarkers show that treatment has changed a biological pathway, such as immune activation during BCG therapy, but they do not necessarily prove tumor clearance.

Each intended use therefore requires separate validation. Diagnostic performance does not establish prognostic value; prognostic association does not demonstrate treatment prediction; pharmacodynamic change does not prove clinical response; and post-treatment molecular positivity should not be called molecular residual disease without appropriate timing, source specificity, and outcome validation. Rather than evaluating urinary biomarkers solely by analyte class or analytical performance, the following sections organize them according to the clinical decisions they are intended to inform. These decision contexts, their corresponding cancer settings, intended test roles, and principal harms to avoid are summarized in Table 2.

## 3. Biological Signals: What Can and Cannot Be Inferred from Urine

Urine contains several layers of cancer-associated information, but these layers do not support equivalent biological claims. A somatic mutation, inflammatory cytokine, collagen fragment, microbial taxon, or EV transcript may differ between patients with and without cancer, yet each reflects a different relationship to the tumor. For clinical interpretation, the key question is not only what is measured, but what level of biological inference the measurement can support.

Four broad levels of inference are useful (Supplementary Table S1). The first and strongest level is evidence of malignant material. This includes tumor-informed mutations, clonal structural alterations, copy-number profiles, unequivocally malignant cytology, and, in selected settings, strongly tumor-associated methylation or RNA signatures. Such findings are most compatible with malignant cells or malignant-cell-derived material entering the sampled urinary pathway [8,17,19,20,21,44]. However, even a tumor-specific alteration does not automatically localize the lesion, quantify viable tumor burden, or distinguish residual disease from newly shed nonviable material. Tumor-informed detection of a patient-specific mutation or structural alteration provides stronger evidence than tumor-naïve panels, but detectability still depends on anatomical access, shedding, specimen fraction, dilution, treatment status, and analytical recovery [35,44]. In bladder cancer, direct luminal shedding creates the most favorable conditions for recovery of malignant material, whereas UTUC, prostate cancer, and RCC add constraints related to upstream transit, collection-dependent enrichment, ductal secretion, renal injury, or systemic circulation [7,19]. A negative result is therefore informative only when access, shedding, fraction suitability, and assay sensitivity are sufficient.

The second level is evidence of an altered epithelial or field state. Histologically nonmalignant epithelium can produce cancer-associated urinary signals, particularly in urothelial carcinoma. Normal-appearing urothelium may contain clonally expanded mutations, methylation changes, or transcriptional programs associated with carcinogen exposure and future tumor development [12,13,23,45,46,47]. Consequently, urinary mutation or methylation positivity after visible tumor removal may reflect residual disease, occult carcinoma in situ, an upper-tract lesion, a persistent molecular field, or regenerative repair. This distinction is clinically important: field positivity is not equivalent to viable tumor, but it is not necessarily biologically irrelevant. Infection, calculi, obstruction, instrumentation, treatment, and post-resection healing can also release nucleic acids, proteins, vesicles, inflammatory mediators, and cellular debris into urine. Longitudinal sampling is therefore required to distinguish transient injury-associated positivity from persistent field alteration or emerging recurrence.

The third level is evidence of tumor–host interaction or treatment engagement. Urinary immune, stromal, vascular, and matrix-remodeling signals may capture immune recruitment, inflammation, angiogenesis, tissue repair, invasion, fibrosis, or treatment-induced biological changes. Single-cell profiling has shown that urine from patients with bladder cancer can contain diverse immune populations, some of which resemble tumor-infiltrating immune cells [22]. However, immune recruitment alone does not establish tumor control. Leukocyturia, cytokine release, neutrophilia, checkpoint-associated signals, or immune-cell EVs may reflect infection, instrumentation, BCG-induced inflammation, epithelial injury, or tumor-associated immune recruitment [48,49,50,51,52]. Similarly, stromal and vascular markers, including VEGF-related factors, TGF-β-related factors, MMPs, TIMPs, collagen fragments, and stromal or endothelial EV cargo, may encode invasion or remodeling, but they also arise during wound healing, renal fibrosis, obstruction, vascular injury, inflammation, and postoperative repair [12,53,54,55]. These signals are therefore best interpreted as mixed tumor–host or pharmacodynamic markers unless paired with tumor-informed molecular, cytological, imaging, or longitudinal outcome evidence [35,36]. During BCG or systemic therapy, a strong immune signal may indicate pharmacodynamic engagement while viable tumor persists; conversely, a declining inflammatory signal may reflect resolution of treatment-induced cystitis rather than tumor response [27,37,49,50].

The fourth level is contextual modification and confounding. Urinary tract infection, cystitis, prostatitis, benign prostatic hyperplasia, calculi, hematuria, chronic kidney disease, proteinuria, obstruction, renal tubular injury, nephron mass, and treatment-related injury can all modify urinary composition and generate cancer-associated but non-malignant signals [7,56,57,58,59,60]. The urinary microbiome may influence inflammation, epithelial turnover, carcinogen metabolism, and response to therapy, but microbiome studies remain limited by low biomass, contamination risk, collection method, sex, geography, antibiotic exposure, sequencing platform, and pipeline variability [46,61]. These contextual factors are not merely technical confounders; they are biological determinants of the sampled compartment.

This inferential hierarchy separates evidence of malignant-cell presence from evidence of a cancer-associated, treatment-modified, or context-modified tissue state. Tumor-informed mutations and malignant cytology can provide relatively direct evidence of tumor material, whereas methylation, transcript, protein, vesicle, immune, stromal, microbial, and renal-associated signatures often encode mixed tumor–host or contextual biology. A urinary result may therefore be biologically genuine yet diagnostically nonspecific. The practical implication is that each urinary signal should be interpreted according to what it can and cannot prove for a defined clinical decision: whether cancer is present, whether invasive evaluation can be deferred, whether disease can be localized, whether recurrence is emerging, or whether treatment has changed tumor or host biology.

## 4. Clinical Decision Contexts for Urinary Biomarkers Across Urological Cancers

This section applies the proposed framework to the principal clinical decision contexts in bladder cancer, UTUC, prostate cancer, and RCC, focusing on what urine can realistically capture in each setting, which clinical decisions urinary biomarkers may inform, and which claims remain insufficiently supported.

### 4.1. Bladder Cancer: Direct Sampling of a Dynamically Perturbed Organ

Bladder cancer is the most mature urinary biomarker setting because malignant urothelium is directly exposed to vesical urine. This anatomical relationship enables recovery of exfoliated tumor cells, tumor DNA, methylated DNA, RNA, proteins, EVs, and immune cells. However, the bladder is also repeatedly affected by hematuria, infection, cystoscopy, TURBT, intravesical therapy, and post-treatment inflammation. Urinary biomarkers in bladder cancer should therefore be interpreted as decision-support tools for a directly sampled but dynamically perturbed organ, rather than as stand-alone replacements for cystoscopy, pathology, or imaging.

#### 4.1.1. Detection in Hematuria Pathways

The clinical question in hematuria evaluation is whether urinary testing can identify patients who require cystoscopy and imaging while safely excluding clinically consequential urothelial carcinoma. The current standard remains cystoscopy, supported by imaging and cytology according to risk. Urine cytology remains the benchmark urinary test because of its high specificity for high-grade urothelial carcinoma and carcinoma in situ, but its sensitivity is substantially lower for low-grade papillary tumors [21,62]. Thus, positive cytology can strongly support high-grade disease, whereas negative cytology cannot reliably exclude malignancy.

The role of biomarkers in this setting is triage rather than definitive diagnosis. Protein-based injury assays, including NMP22 and BTA, may improve sensitivity compared with cytology in some cohorts, but their specificity is affected by epithelial injury, bleeding, inflammation, infection, stones, and recent instrumentation [21,63]. Mutation-based assays detect tumor-associated genomic alterations, whereas methylation-based assays capture broader epigenetic states that may occur across tumor grades and in molecularly altered urothelium [64,65]. Transcriptomic and composite classifiers integrate molecular features, and sometimes clinical variables, to improve detection and risk stratification [66].

The maturity of the evidence is highest for bladder cancer compared with other urological cancers, but clinical implementation still depends on decision-specific validation. For hematuria pathways, the unacceptable harm is missed high-grade urothelial carcinoma or delayed diagnosis. Therefore, prospective hematuria-positive cohorts, including benign inflammatory and bleeding conditions, are more relevant than case–control discrimination alone. A biomarker intended to reduce cystoscopy must demonstrate consistently high negative predictive value for high-grade disease and an explicitly acceptable miss rate.

Reported sensitivity, specificity, positive predictive value (PPV), and negative predictive value (NPV) should be interpreted within the intended clinical setting rather than compared directly across assays, because these estimates vary with disease prevalence and spectrum, tumor grade, specimen characteristics, assay thresholds, and reference standards. Accordingly, this review emphasizes decision-specific performance and clinical utility rather than cross-study ranking of urinary assays.

#### 4.1.2. Residual Disease After TURBT

The clinical question after TURBT is whether a urinary biomarker can distinguish complete tumor removal from residual microscopic disease, occult carcinoma in situ, or early molecular recurrence. The current standard is pathological assessment of the resected tumor, adequacy of resection, risk stratification, repeat TURBT when indicated, cystoscopy, and cytology. Urinary biomarkers may add value if they identify persistent tumor-derived material after an appropriate post-procedural interval.

Tumor-informed urinary DNA has the strongest biological rationale in this setting. Detection of patient-specific mutations after apparently complete resection provides stronger evidence of clonal persistence than generic protein, inflammatory, or transcript elevation [35,67]. Conversely, mutation clearance or marked decline may support effective tumor removal, although false-negative results can arise from low shedding, inappropriate specimen fractions, insufficient assay sensitivity, or panel mismatch. Complementary evidence from circulating tumor DNA in muscle-invasive bladder cancer further supports the broader potential of tumor-derived DNA for molecular residual disease assessment, prognostic stratification, and treatment-response monitoring, particularly when evaluated longitudinally in relation to systemic therapy and clinical outcomes [68].

Methylation persistence is more difficult to interpret. Cancer-associated methylation may remain detectable because of viable residual tumor, occult carcinoma in situ, or a molecularly altered urothelial field that persists after visible tumor removal [12,69]. Such persistence may be prognostically informative, but it does not by itself prove that viable malignant cells remain.

The unacceptable harm in this setting is misclassifying acute post-resection release or field alteration as molecular residual disease, or conversely missing persistent high-risk disease. Timing is therefore central. Molecular positivity immediately after TURBT may reflect blood, disrupted urothelium, necrotic material, inflammatory mediators, and procedure-related DNA release. Post-washout persistence or re-emergence after initial clearance is more compatible with residual clonal disease, occult carcinoma in situ, or field-related abnormality, particularly when the signal is tumor informed. Studies should therefore prespecify sampling intervals and distinguish acute procedural release from persistent or re-emerging tumor-derived signal.

#### 4.1.3. Recurrence Surveillance

The clinical question in surveillance is whether urinary biomarkers can reduce, defer, or prioritize cystoscopy without missing clinically consequential recurrence. The current standard remains scheduled cystoscopy, often supported by cytology and risk-adapted surveillance intervals. Biomarkers are most clinically plausible as cystoscopy-triage tools, particularly when they achieve high negative predictive value for high-grade recurrence.

Cxbladder Monitor, Xpert Bladder Cancer Monitor, Bladder EpiCheck, Uromonitor, and related assays have reported high negative predictive values, particularly for high-grade recurrence [66,70,71]. Their role should therefore be framed as surveillance support rather than independent confirmation of recurrence. A negative biomarker may help identify lower-risk intervals or support deferral strategies only if validated prospectively within a defined surveillance pathway.

Serial molecular testing may also detect recurrence before lesions become visible endoscopically. Urinary mutation and methylation signals have anticipated cystoscopic or clinical recurrence in longitudinal studies, creating a state of molecular anticipation [67,69]. However, biomarker-positive and cystoscopy-negative findings are biologically heterogeneous: they may represent occult carcinoma in situ, a small papillary lesion, upper-tract disease, persistent field alteration, procedure-related injury, or a false-positive assay result.

The unacceptable harm is false reassurance leading to delayed detection of high-grade or progressive disease. Longitudinal trajectories are therefore more informative than isolated measurements. Persistent positivity, failure to clear after resection, or a rising molecular signal may carry greater clinical meaning than a single low-level positive result. However, evidence that intervention during a molecularly positive but endoscopically negative state improves outcomes remains limited.

#### 4.1.4. BCG-Response Monitoring

The clinical question during intravesical BCG therapy is whether urinary biomarkers can distinguish immune engagement, treatment toxicity, molecular tumor clearance, and true clinical response. The current standard remains cystoscopy, cytology, biopsy when indicated, and risk-adapted assessment of recurrence or progression. Urinary biomarkers may be useful if they clarify whether BCG has activated the intended immune pathways and whether tumor-derived signals are clearing.

BCG creates a deliberately perturbed urinary immune compartment. Baseline immune context may influence response, whereas early post-instillation samples capture acute innate activation dominated by granulocytes, inflammatory cytokines, and myeloid-cell recruitment [72]. Later measurements may reflect adaptive engagement, including T-cell and chemokine responses. These immune states are biologically informative but should not be equated with tumor eradication.

The biomarker role is therefore partly pharmacodynamic and partly molecular. Immune engagement indicates activation of local immune pathways, whereas immune toxicity reflects excessive or symptomatic inflammation. Molecular tumor clearance requires the decline or disappearance of tumor-informed DNA, RNA, cytological, or other malignant-cell-associated signals. Clinical response refers to the absence of visible or histologically confirmed recurrence or progression. These outcomes can overlap, but they are not interchangeable.

The CyPRIT model, which integrates changes in nine urinary cytokines, illustrates that BCG response may be encoded in coordinated kinetics rather than single measurements [73]. Sustained CXCL10 release has likewise been associated with BCG-induced immune engagement [74]. Nevertheless, increased inflammation is not necessarily beneficial, and cytokine induction alone should not be treated as proof of tumor clearance.

The unacceptable harm is either premature escalation based on inflammation alone or delayed treatment change in a patient with persistent tumor biology. BCG-response monitoring should therefore integrate immune pharmacodynamics with tumor-informed molecular clearance, cytology, cystoscopy, and clinical outcomes.

#### 4.1.5. Progression and Escalation Decisions

The clinical question in progression and escalation is whether urinary biomarkers can identify patients who require repeat TURBT, intensified surveillance, alternative intravesical therapy, systemic therapy, or early radical cystectomy. The current standard remains clinicopathological risk stratification, histology, grade, stage, CIS status, response to BCG, cystoscopy, cytology, imaging, and multidisciplinary decision-making.

The potential biomarker role is risk refinement and escalation support. Persistent tumor-informed urinary DNA, failure of a molecular signal to clear after resection or treatment, and high-grade cytology have been associated with residual disease, recurrence, or adverse pathological findings [35,67,69]. Combining persistent tumor-derived signals with suppressive immune features, such as myeloid-derived suppressor-cell enrichment or exhausted urinary T-cell states, may further improve identification of patients at risk of BCG failure [22,72].

However, the evidence maturity remains insufficient for urinary biomarkers to independently determine escalation. Most available evidence is observational and has not established that biomarker-guided repeat resection, alternative intravesical therapy, or radical cystectomy improves survival or safely reduces treatment burden [21,66]. The unacceptable harm is overtreatment, including unnecessary cystectomy, or undertreatment of biologically aggressive disease. Until prospective interventional trials define validated thresholds and management algorithms, urinary biomarkers should complement—not replace—pathology, cystoscopy, imaging, and established clinicopathological risk models.

#### 4.1.6. Translational Readiness

Bladder cancer has the most mature evidence base for urinary biomarkers among urological malignancies. Cytology remains the established urinary adjunct to cystoscopy, particularly for high-grade disease, while molecular assays including Cxbladder, Xpert Bladder Cancer Monitor, Bladder EpiCheck, and Uromonitor have accumulated substantial validation data for NMIBC detection and surveillance [66]. Mutation- and methylation-based assays, including urine tumor DNA approaches, further show potential for recurrence detection and longitudinal monitoring [67,69]. However, cystoscopy remains the reference standard, and broader implementation requires greater preanalytical standardization [31], clearer management of biomarker-positive/cystoscopy-negative findings, and prospective evidence that biomarker-guided surveillance can safely reduce invasive procedures.

### 4.2. Upper-Tract Urothelial Carcinoma: Direct Contact Modified by Distance and Collection Site

UTUC arises from the urothelium of the renal pelvis or ureter and therefore has direct luminal contact with urine. However, unlike bladder cancer, the lesion is located upstream from the final voided specimen. Tumor-derived material must traverse the ureter, undergo dilution, mix with bladder and lower-tract material, and remain sufficiently intact for detection [75,76]. UTUC urinary biomarkers should therefore not be presented as equivalent to bladder cancer biomarkers, because anatomical localization, specimen access, and clinical evidence maturity are substantially different.

#### 4.2.1. High-Grade Disease Detection

The main clinical question is whether urinary testing can identify high-grade UTUC that requires ureteroscopy, biopsy, or definitive treatment. Distance between shedding and collection is central to this question. Voided urine generally has lower sensitivity than selectively collected renal-pelvic or ureteral urine [77,78,79]. Selective cytology obtained close to the lesion can achieve substantially higher sensitivity than voided cytology, supporting the importance of proximity and reduced downstream dilution [80]. A single negative voided specimen therefore cannot exclude disease; in contemporary series, urine cytology alone missed a substantial proportion of pathologically confirmed high-grade UTUC [81].

Obstruction and hydronephrosis further complicate detection. A tumor may retain cells and nucleic acids proximal to an obstruction while simultaneously increasing epithelial injury, inflammation, hematuria, and tubular-stress signals [33,82]. Thus, a negative voided test may reflect poor downstream access rather than absence of disease, whereas a positive signal may reflect tumor, injury, or inflammation.

Urine cytology remains central to UTUC evaluation because a clearly positive result is highly specific for high-grade urothelial carcinoma, although sensitivity is substantially lower for low-grade tumors [83,84]. Selective upper-tract cytology generally provides greater diagnostic relevance than voided cytology, particularly when cystoscopy has excluded bladder disease. The Paris System improves standardization by emphasizing high-grade disease, but instrumentation-related atypia and low cellularity remain important limitations [85,86].

FISH provides a cell-based measure of chromosomal abnormality and can improve sensitivity relative to cytology in some UTUC cohorts [87,88]. Mutation assays provide a more direct measure of tumor-associated genomic material, and targeted urinary DNA panels have shown diagnostic potential in UTUC, particularly when combined with cytology [89]. Methylation assays can detect cancer-associated epigenetic states, including in tumors without informative targeted mutations, and paired studies have reported higher sensitivity than cytology for some methylation panels, including in high-grade disease [90,91]. However, neither FISH nor molecular positivity in voided urine reliably localizes the lesion, and methylation positivity may reflect visible tumor, premalignant clones, field cancerization, inflammation, or previous bladder cancer.

Integrated urinary tumor DNA approaches combining genomic and epigenomic features may eventually contribute to preoperative risk stratification. Broad genomic instability or high-risk molecular profiles may support suspicion of aggressive disease, but urinary genotype cannot reliably determine depth of invasion. These assays should therefore complement, rather than replace, imaging, ureteroscopic biopsy, tumor size, multifocality, cytology, and hydronephrosis when deciding between kidney-sparing treatment and radical nephroureterectomy [76,92].

#### 4.2.2. Selective Urine Versus Voided Urine

The most important sampling distinction in UTUC is selective upper-tract urine versus voided urine. Selective renal pelvic or ureteral urine improves anatomical attribution because it is obtained closer to the suspected lesion, but it requires catheterization or ureteroscopy and may introduce saline dilution, bleeding, epithelial trauma, and low specimen volume. Voided urine is easier to collect but cannot reliably distinguish upper-tract disease from bladder recurrence, synchronous bladder cancer, or a pan-urothelial field effect. Selective and voided urine should therefore be considered distinct sampling compartments, not interchangeable specimens.

#### 4.2.3. Kidney-Sparing Treatment Surveillance

Kidney-sparing treatment leaves the ipsilateral upper tract in place and creates a need for repeatable biomarkers capable of detecting residual disease, local recurrence, progression, contralateral disease, and subsequent bladder cancer. Current surveillance remains dependent on ureteroscopy, imaging, cystoscopy, and cytology because no urinary assay has demonstrated sufficient sensitivity, localization, and clinical-outcome benefit to replace these procedures [92,93].

After endoscopic ablation, a positive selective upper-tract assay has greater anatomical plausibility for ipsilateral residual or recurrent disease than a positive voided test. Tumor-informed urinary DNA may be particularly valuable when the detected alteration matches the index tumor. However, ureteroscopy, laser ablation, stenting, and topical therapy can cause hematuria, epithelial injury, inflammatory recruitment, and release of nonviable tumor DNA. Sampling time must therefore be specified before a post-treatment signal is interpreted as molecular residual disease.

Post-treatment urinary signals should be interpreted by phase: acute procedural release, washout, post-washout persistence, and re-emergence after clearance. Persistent or recurrent tumor-informed positivity is more concerning than immediate post-procedure positivity, but anatomical confirmation remains necessary because voided urine may reflect ipsilateral UTUC, contralateral disease, bladder recurrence, or field alteration. Bladder recurrence after UTUC treatment may arise through intraluminal seeding, shared clonal evolution, or independent tumor development within a pan-urothelial field [94,95]. Comparison with the original tumor genotype, side-specific selective sampling, negative cystoscopy, and longitudinal clearance after local treatment can strengthen attribution, but voided urine alone rarely provides anatomical certainty.

#### 4.2.4. Localization as the Central Translational Barrier

The defining translational challenge in UTUC is localization. A positive voided biomarker may indicate active upper-tract disease, synchronous or recurrent bladder cancer, pan-urothelial field alteration, residual nonviable tumor material, or treatment-related epithelial injury. These states require different clinical responses.

Accordingly, voided urinary biomarkers are most credible as triage tools that guide the urgency and direction of imaging, ureteroscopy, or selective sampling. Tissue–urine genomic concordance, prior tumor sequencing, selective sampling, and longitudinal dynamics can strengthen attribution. For UTUC, anatomical attribution is not a secondary analytical consideration; it is a prerequisite for clinical translation.

#### 4.2.5. Translational Readiness

Urinary biomarkers for UTUC are less mature and more dependent on sampling site than those for bladder cancer. Selective upper-tract cytology remains the most established urine-based test, with FISH serving as an adjunctive cellular assay [84,85,86,87,88,92]. Molecular approaches, including urinary mutation and DNA methylation assays, have shown promising diagnostic performance but remain investigational [89,90,91]. Translation is limited by variable tumor shedding, dilution and obstruction, instrumentation-related effects, limited prospective validation, and the inability of a positive voided-urine signal to reliably localize disease to the upper urinary tract [75,78,82,84].

### 4.3. Prostate Cancer: Decision-Support Testing for Clinically Significant Disease

Prostate cancer differs from urothelial malignancies because most localized tumors do not contact the urinary lumen directly. Prostate-associated material reaches urine through glandular secretion, prostatic ducts, epithelial exfoliation, and EV release into the urethra, making the urinary signal strongly dependent on collection strategy [18,19]. First-catch urine preferentially captures urethral and prostate-derived material, whereas DRE can further increase the release of prostate-derived cells, RNA, proteins, and vesicles. PCA3, SelectMDx, and the original MyProstateScore were developed using post-DRE collection, whereas ExoDx Prostate and more recent MyProstateScore 2.0 protocols can use first-catch urine without mandatory manipulation [40,96,97]. These assays should therefore be interpreted as prostate-enriched decision-support tests, not as general cancer-detection tools.

Enrichment of prostate origin does not equal enrichment of tumor origin. Benign prostate epithelium contributes abundant glandular transcripts and EVs, and recovered signals may vary with prostate volume, secretory activity, inflammation, recent manipulation, and DRE technique [18,98,99,100]. Prostate-volume-adjusted models and normalization to prostate-reference transcripts such as KLK3 can partly address variable prostate-cell recovery, but they cannot fully remove variation related to gland size, inflammation, collection technique, or differential secretion [99,101,102]. Histopathological inflammation has also been associated with systematic changes in urinary PCA3 scores, and urinary EVs may originate from multiple benign and malignant sources despite their potential to preserve RNA and partial cell-of-origin information [98,103].

#### 4.3.1. Pre-Biopsy Triage

The principal clinical use of urinary biomarkers in prostate cancer is not detection of every malignant focus, but enrichment for clinically significant disease, usually defined as GG2 or higher, among men being considered for biopsy [40,96,97]. The current standard pathway combines serum PSA, PSA density, clinical risk calculators, MRI, and shared decision-making. PSA is sensitive to prostatic pathology but lacks cancer specificity because it is influenced by benign prostatic hyperplasia, prostatitis, gland volume, and recent manipulation [104,105]. MRI improves risk assessment, but negative or equivocal imaging does not fully exclude GG2 or higher cancer, particularly in men with PI-RADS 3 lesions or persistent clinical suspicion [105,106].

Urinary assays are best positioned as biopsy-triage tools. PCA3 established the feasibility of measuring prostate-derived RNA in post-DRE urine and improved repeat-biopsy risk assessment compared with PSA alone [43,107]. However, PCA3 alone has limited ability to distinguish indolent from clinically significant cancer and does not consistently provide the sensitivity or negative predictive value required for contemporary GG2-or-higher triage [107,108]. Its current role is therefore molecular risk enrichment, particularly within multivariable or multigene models [43,97].

Multigene RNA and EV-based classifiers aim to improve detection of clinically significant disease [109]. The HOXC6–DLX1 signature used in SelectMDx was developed to enrich for high-grade cancer and may reduce unnecessary biopsy at high-sensitivity thresholds [40]. The original MyProstateScore combined serum PSA with urinary PCA3 and TMPRSS2:ERG, whereas MyProstateScore 2.0 uses an expanded transcript panel optimized for GG2 or higher cancer [43,97]. ExoDx Prostate measures EV RNA in first-catch urine without mandatory DRE and has shown high sensitivity and negative predictive value for GG2 or higher disease in prospective validation [96].

#### 4.3.2. Integration with MRI

RI and urinary biomarkers provide complementary information. Multiparametric MRI evaluates lesion location, morphology, volume, diffusion restriction, and contrast-enhancement patterns, whereas urinary assays measure prostate-associated molecular expression or EV cargo. Their integration may be most useful when MRI is negative or equivocal [105,110].

Three clinical states are especially relevant. First, MRI-positive and urine-high-risk findings provide concordant anatomical and molecular evidence, strengthening the indication for biopsy. Second, MRI-equivocal and urine-high- or low-risk findings are particularly important for PI-RADS 3 lesions. A meta-analysis of 56 studies reported a pooled positive predictive value of approximately 13% for clinically significant prostate cancer in PI-RADS 3 lesions, supporting individualized risk assessment [111]. Urinary MyProstateScore remained independently associated with GG2 or higher cancer across PI-RADS categories and outperformed PSA density for ruling out GG2 or higher disease in men with PI-RADS 3 findings [112]. In this context, a low-risk urinary result may support biopsy deferral, whereas a high-risk result may strengthen the indication for biopsy within a prespecified pathway.

Third, MRI-negative and urine-high-risk discordance may reflect clinically significant disease below MRI resolution, small-volume or diffuse molecularly abnormal cancer, or a false-positive molecular signal from benign glandular or inflammatory biology. Retrospective pathway analyses combining ExoDx or other liquid biomarkers with MRI reduced unnecessary biopsies, but the number of missed GG2-or-higher cancers depended strongly on test order and thresholds [110,113]. Discordance should therefore prompt pathway-defined reassessment, repeat imaging, systematic or targeted biopsy, or surveillance rather than automatic escalation.

The clinically relevant endpoint is safe biopsy avoidance. Studies should report biopsies avoided, GG2-or-higher cancers missed, calibration, and decision-curve net benefit. In a prospective multicenter study, SelectMDx alone avoided 38% of biopsies while missing 10% of high-grade cancers; an MRI-first strategy avoided 49% while missing 4.9%, whereas a conditional SelectMDx-first strategy reduced MRI use but missed more high-grade cancers [114]. Prospective comparative studies are still needed to define whether urinary testing is best used before MRI, after equivocal MRI, after negative MRI with persistent suspicion, or within an integrated multivariable model.

#### 4.3.3. Active Surveillance and Upgrading

Urinary biomarkers have an emerging but not established role in active surveillance. The clinical question is whether they can identify occult higher-grade disease or increased reclassification risk in men initially managed without definitive treatment. This is clinically relevant because systematic and targeted biopsies sample only part of the prostate and may miss spatially separate higher-grade foci or underestimate intratumoral heterogeneity [115,116].

Early evidence suggests that urinary EV transcript profiles may help identify active-surveillance patients at risk of reclassification [117]. However, this evidence remains preliminary and should be distinguished from the stronger evidence base for initial biopsy triage. Expanded urine RNA models such as MPS2 have so far been validated primarily for pre-biopsy detection of GG2-or-higher cancer rather than for serial surveillance or upgrade prediction [97,101]. Therefore, a score that predicts GG2-or-higher disease at diagnostic evaluation should not be assumed to predict longitudinal upgrading, biological progression, or timing of repeat biopsy. MRI- and PSA-density-guided strategies remain the principal comparators for future urinary-biomarker studies in active surveillance [115,116,118].

Longitudinal changes in urinary scores may reflect true molecular progression or increasing tumor contribution, but may also arise from prostatitis, altered benign glandular secretion, prostate manipulation, specimen variability, or assay imprecision. Implementation requires prospective evidence that serial biomarker trajectories predict GG upgrading beyond baseline pathology, PSA kinetics, PSA density, and MRI progression [116,118]. Studies must also define actionable thresholds and show that biomarker-triggered repeat MRI or biopsy reduces procedural burden without delaying detection of clinically significant disease.

Urinary immune monitoring remains exploratory in prostate cancer. Unlike bladder cancer, the prostate tumor–immune interface does not directly shed into urine, and urinary cytokines or immune-related transcripts may arise from prostatitis, benign prostatic hyperplasia, urethral cells, urinary infection, or systemic sources [104]. Until paired tissue–urine studies show that urinary immune markers reproducibly reflect intratumoral immune biology rather than benign prostatic inflammation, they should not be used for clinical decision-making.

#### 4.3.4. Translational Readiness

Urinary biomarkers in prostate cancer are most clinically advanced for pre-biopsy risk stratification. Following the development of PCA3, newer assays including SelectMDx, ExoDx Prostate, MyProstateScore (MPS), and MPS2 have increasingly focused on identifying clinically significant, GG ≥ 2 disease while reducing unnecessary biopsy [39,40,96,97,99,101]. Several strategies combining urinary biomarkers with MRI and other clinical risk parameters have been evaluated, but the optimal sequencing and integration of these tools within diagnostic pathways remain to be established [110,112,113,114]. Evidence for urinary biomarkers in active surveillance and longitudinal disease monitoring is considerably less mature, although emerging extracellular-vesicle approaches suggest potential for detecting risk reclassification [108,109,117]. Wider implementation is limited by heterogeneity in urine collection and assay methodology, contributions from benign and inflammatory prostatic processes, and limited prospective evidence that biomarker-guided diagnostic pathways improve clinically meaningful outcomes [18,19,100,103,108,109].

### 4.4. Renal Cell Carcinoma: Unresolved Urinary Source Attribution

RCC presents a liquid-biopsy paradox: it arises within the organ that produces urine, yet most renal tumors do not communicate directly with the urinary collecting system. Clear-cell and papillary RCC usually originate from renal tubular epithelial lineages within the cortex, separated from the final urinary stream by renal parenchyma, tubular architecture, and the collecting system [54,119]. Urinary signals in RCC may therefore arise from several non-equivalent sources, including tumor communication with the collecting system, hematuria, renal epithelial injury, tubular secretion, EV transport, glomerular filtration, systemic inflammation, or host-response biology.

Direct communication with urine can occur when centrally located or locally advanced RCC invades the renal pelvis, calyces, or collecting system [120,121,122]. However, this mechanism is unlikely to represent most small, incidentally detected cortical masses and should not be assumed solely because the tumor is located in the kidney [20,119]. Hematuria can introduce erythrocytes, leukocytes, plasma proteins, circulating nucleic acids, and EVs into urine, but these components cannot automatically be attributed to tumor because stones, infection, anticoagulation, benign renal masses, and non-neoplastic kidney disease can produce similar contamination [8,122,123,124]. Renal epithelial release adds further ambiguity: normal, injured, and malignant tubular epithelial cells can all release proteins, metabolites, RNA, and vesicles into urine. Urinary aquaporin-1 and perilipin-2 decrease after nephrectomy, supporting a tumor-bearing-kidney association, but not eliminating the contribution from adjacent non-malignant renal tissue [125,126]. Similarly, most urinary EVs originate from non-malignant nephron and urinary-tract epithelium, and urinary recovery of circulating proteins or cell-free DNA reflects filtration, tubular handling, local release, and degradation rather than tumor production alone [127,128,129,130].

The most plausible clinical opportunity is characterization of incidentally detected renal masses. A clinically useful urinary test would need to distinguish malignant RCC from oncocytoma, angiomyolipoma, complex cystic lesions, and non-neoplastic renal pathology, not simply separate RCC from healthy controls. Urinary proteins currently provide the most practical foundation. Aquaporin-1 and perilipin-2 have shown promise for detecting clear-cell and papillary RCC and for characterizing small renal masses, but reduced expression in chromophobe and oncocytic tumors creates a subtype-specific false-negative risk [126,131]. Urinary metabolomic signatures are biologically attractive because clear-cell RCC is characterized by VHL loss, HIF activation, pseudohypoxia, lipid accumulation, altered redox biology, and mitochondrial metabolic rewiring [132,133]. Several studies have reported RCC-associated urinary metabolomic profiles, including discrimination by stage or separation from oncocytoma [134,135,136]. However, urinary metabolites are strongly modified by diet, hydration, medications, diabetes, obesity, microbiome composition, glomerular filtration, and tubular handling; classifiers therefore require validation in renal-mass cohorts with benign tumors and relevant comorbidities [8,137].

Urinary DNA, methylation, RNA, and EV-based assays remain in earlier stages of development. Urinary cell-free DNA may contain RCC-associated mutations, copy-number alterations, fragmentomic features, or methylation patterns, but mutation-based detection is constrained by low shedding from localized RCC [20]. Methylation-based approaches may provide broader signal coverage, and cell-free methylome analysis has distinguished RCC from controls in both plasma and urine, although performance has generally been stronger in plasma [138]. Urinary EV RNA signatures have shown promising discrimination of early-stage RCC, but external validation, source attribution, isolation standardization, and normalization remain limited [139,140]. Across these domains, the unresolved question is whether the signal reflects malignant tissue rather than renal injury, benign renal epithelium, hematuria, or systemic biology.

Treatment monitoring is a future research opportunity rather than a validated clinical use. A credible postoperative urinary recurrence marker would need to be detectable before surgery, decline after tumor removal beyond changes caused by nephron loss or perioperative injury, and reappear before or with radiological recurrence. During systemic therapy, urinary profiling could in principle track angiogenic, immune, metabolic, or kidney-injury signals [54,119]. However, VEGF-pathway inhibitors can cause proteinuria, endothelial injury, podocyte dysfunction, and thrombotic microangiopathy, whereas immune-checkpoint inhibitors can cause tubulointerstitial nephritis and immune-mediated glomerular or tubular injury [141,142]. Urinary changes during treatment may therefore reflect tumor response, nephrotoxicity, altered renal physiology, or all three.

RCC urinary biomarkers should therefore be presented as exploratory and hypothesis-generating. Urinary proximity to the kidney does not guarantee tumor specificity. Candidate biomarkers should be validated not only against healthy individuals but also against benign renal masses, chronic kidney disease, non-malignant hematuria, urinary infection, diabetes-related nephropathy, and other causes of tubular injury [8]. A urinary analyte should not be described as tumor-derived until renal physiological alternatives have been explicitly evaluated through tissue concordance, renal-function adjustment, perioperative kinetics, parallel plasma analysis, and longitudinal association with recurrence or treatment response.

#### Translational Readiness

Urinary biomarkers for RCC remain largely investigational, with no assay currently established for routine renal-mass characterization, postoperative surveillance, or treatment monitoring [7,8,55]. AQP1 and PLIN2 are among the better-studied candidate urinary proteins, including prospective evaluation and studies of small renal masses [126,131], while metabolomic [134,135,136,137], epigenetic and cell-free DNA [20,138], non-coding RNA [139], microRNA, and extracellular-vesicle signatures [140] remain investigational. Translation is constrained by the biological complexity of urinary signal origin and by potential contributions from renal parenchymal and tubular processes, renal dysfunction, hematuria, and non-malignant renal disease [127,128]. Prospective validation in representative renal-mass cohorts, with appropriate consideration of renal function and benign renal pathology, paired tissue–urine analyses, and longitudinal sampling, is therefore required before clinical implementation [7,8].

## 5. Interpreting Positive, Negative and Discordant Urinary Results

Urinary biomarkers should not be interpreted through a simple tumor-present versus tumor-absent framework. A positive result may indicate viable residual disease, occult unsampled cancer, molecularly altered epithelium, treatment-selected clones, ineffective inflammation, or transient release of nonviable material. Conversely, a negative result may reflect true clearance, low-volume disease, inadequate shedding, anatomical separation from urine, assay-target mismatch, inappropriate specimen fraction, or sample degradation. These possibilities differ across cancers because urinary observability is highest in bladder cancer, limited by dilution and localization in UTUC, dependent on collection strategy in prostate cancer, and least source-specific in RCC (Supplementary Figure S1; Supplementary Table S2).

### 5.1. Interpreting a Positive Urinary Result

A positive urinary result is most clinically persuasive when it is tumor informed, persistent, anatomically plausible, and temporally appropriate. In bladder cancer, persistent or re-emerging urinary tumor DNA after TURBT or intravesical therapy has been associated with residual disease, recurrence, and longitudinal disease status [36,67,143]. Malignant cytology can provide complementary cellular evidence, although it is less molecularly specific. In UTUC, tumor-matched DNA in ipsilateral selective urine after kidney-sparing treatment would provide stronger evidence of residual upper-tract disease than the same alteration in voided urine, but prospective evidence linking post-ablation urinary persistence to recurrence remains limited and surveillance still depends on ureteroscopy, cytology, and imaging [91,144]. In prostate cancer, a high-risk urinary RNA score after a negative or low-grade biopsy indicates increased probability of unsampled GG2-or-higher disease rather than classical postoperative residual disease [101]. In RCC, tumor-informed plasma or urinary DNA can occasionally persist after nephrectomy and track disease course, but RCC is a low-shedding malignancy and urine detection is inconsistent; postoperative proteins and EVs are additionally confounded by nephron loss and tubular injury [20].

Positive signals may also reflect field-level susceptibility rather than localized visible tumor. In urothelial cancer, morphologically normal urothelium may harbor cancer-associated mutations and clonal expansions, creating a recurrence-prone field that can release molecular material despite negative cystoscopy [45,145]. Paired genomic studies indicate that many bladder tumors arising after UTUC are clonally related to the upper-tract primary, although independent primaries also occur [146]. In prostate cancer, low-level molecular positivity is more often explained by benign glandular expression, BPH, prostatitis, or sampling variability than by a defined pan-prostatic field state [101,103]. In RCC, persistent renal epithelial, protein, RNA, or EV signals more plausibly reflect chronic kidney disease or tubular injury than a defined cancer-field state [8,146,147,148]. Thus, positive urinary results can indicate a clinically relevant abnormal compartment, but they do not always prove viable or anatomically localized tumor.

### 5.2. Interpreting a Negative Urinary Result

A negative urinary biomarker result should not automatically be interpreted as absence of disease. True biological negativity is the strongest interpretation, but it requires evidence that the assay can detect the relevant tumor state when present [35,36,143]. Anatomical non-access is common when disease does not communicate sufficiently with the sampled urine, especially in prostate cancer, RCC, obstructed UTUC, or anatomically separated lesions [11,17,20]. Biological non-shedding can occur when a tumor releases little of the targeted analyte because of low volume, low turnover, limited necrosis, unfavorable fraction distribution, or low abundance of the assayed alteration [15,25]. Temporal negativity may reflect sampling during an intermittent or low-release period [143].

Fraction mismatch and analytical failure provide additional explanations. A signal may be enriched in exfoliated cells but missed in supernatant, or present as cell-free DNA, soluble protein, or EV cargo but not captured by a sediment-based assay [25,31]. Delayed processing, analyte degradation, inhibitory urine chemistry, dilution, poor cellularity, insufficient input material, or inadequate assay sensitivity can also produce a negative result [31]. De-escalation based on a negative urinary result should therefore require evidence of anatomical access, shedding sensitivity, fraction suitability, and analytical recovery in the intended tumor state.

### 5.3. Biomarker-Positive but Imaging- or Endoscopy-Negative Results

A biomarker-positive but cystoscopy-, ureteroscopy-, MRI-, biopsy-, or imaging-negative result should not automatically be labeled assay failure. It may represent molecular lead time, subclinical disease below the resolution of the reference test, field alteration, anatomical misattribution, or post-treatment persistence. Urinary tumor DNA or methylation can become positive before cystoscopic bladder recurrence, and tumor-associated urine signals may occur when ureteroscopy, MRI, or biopsy is negative or equivocal [67,143]. Post-treatment persistence can also reflect DNA or RNA released from nonviable cells, surgical bleeding, epithelial injury, treatment-induced necrosis, delayed clearance, BCG-related inflammation, ureteroscopic injury, or post-nephrectomy tissue injury [143,145,149].

The appropriate response is sequential interpretation rather than automatic escalation. Clinicians should verify specimen quality and treatment timing, determine whether the marker is tumor informed, identify plausible anatomical sources, examine serial trajectories, reconsider the sensitivity of the reference standard, and ask whether the result has a validated management threshold (Supplementary Table S2). In urothelial cancer, mutation- or methylation-positive urine with negative cystoscopy may reflect occult CIS, upper-tract disease, a recurrence-prone field, or intraluminal seeding [45,146]. In prostate cancer, a high-risk urinary score with negative or equivocal MRI may reflect MRI-invisible GG2-or-higher disease, spatial undersampling, or a benign molecular source [92,101,105]. Intervention during this discordant state requires prospectively validated action thresholds rather than molecular positivity alone.

### 5.4. Biomarker-Negative but Clinically Suspicious Results

A biomarker-negative result cannot overrule a positive or strongly suspicious clinical assessment. Poor or heterogeneous shedding may produce negative urine despite tumor detected by cystoscopy, imaging, MRI, ureteroscopy, or biopsy. This occurs with small or low-grade bladder tumors, obstructed UTUC, MRI-visible or biopsy-proven prostate cancer with a low-risk urinary score, localized RCC with low urinary ctDNA release, or tumors lacking the assay target [20,101,144]. The correct interpretation is not necessarily that the biomarker is analytically flawed, but that the specimen and assay may not represent the tumor state being tested.

Treatment state can further complicate negative or changing results. BCG-induced inflammation, chemotherapy-mediated cell death, treatment-selected clonal expansion, androgen-pathway effects on prostatic secretion, VEGF-inhibitor nephrotoxicity, immune-checkpoint-inhibitor nephritis, and treatment-related changes in filtration or shedding can all alter urinary signal magnitude without matching clinical response [72,145,149]. Treatment-naïve thresholds should therefore not be transferred uncritically to on-treatment samples. Urinary results should be interpreted relative to pretreatment baseline and alongside imaging, cystoscopy or ureteroscopy, biopsy when indicated, urinalysis, renal function, tumor-informed molecular measurements, and—in RCC or systemic disease—paired plasma analysis.

Overall, urinary biomarkers are most useful when interpreted as decision-support tools within defined clinical pathways. Positive results require source attribution, temporal interpretation, and actionability; negative results require evidence of access and assay adequacy; and discordant results require structured reassessment rather than automatic classification as false positive or false negative. The detailed cross-cancer state model and discordance framework are provided in Supplementary Tables S1–S3, and Supplementary Figure S1 [150,151,152,153,154,155,156,157].

## 6. Practical Evidence Standards for Clinical Translation of Urinary Biomarkers

**Figure 3:**
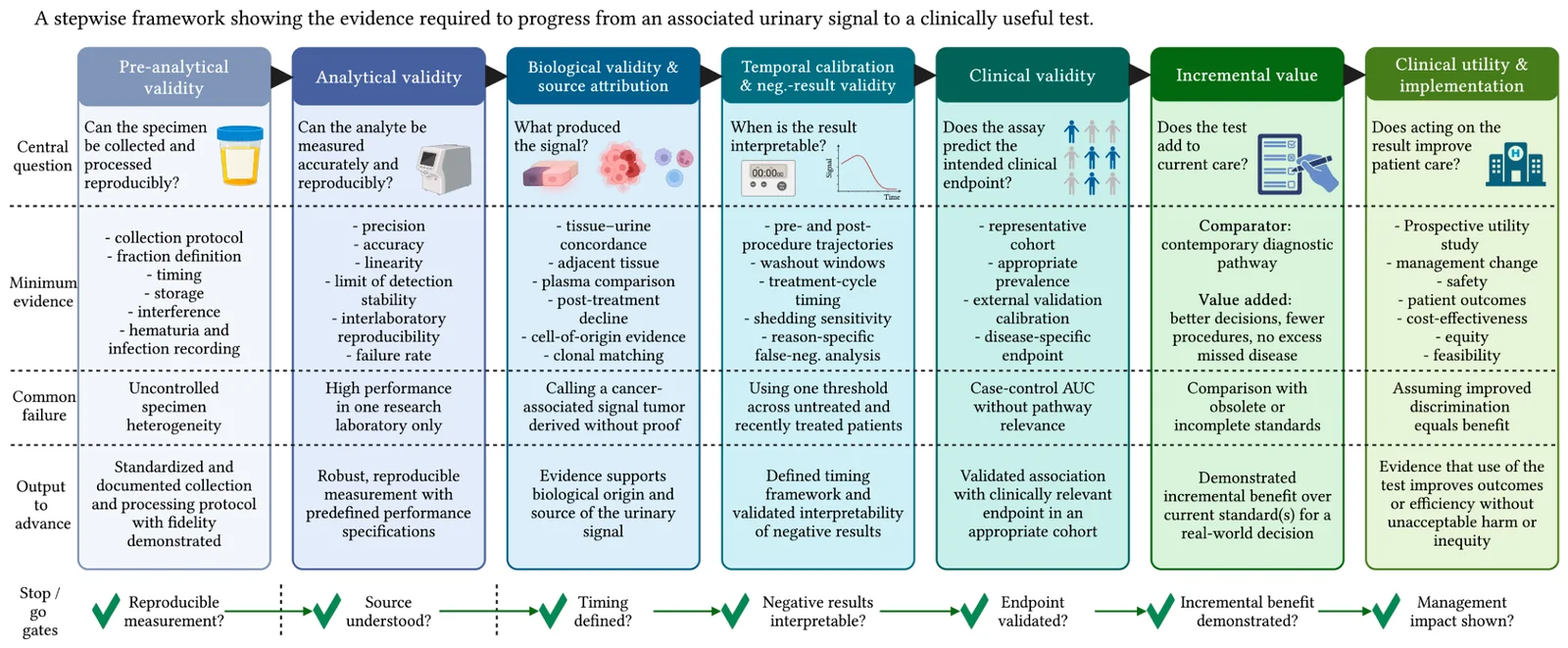
Translational evidence pathway from urinary biomarker discovery to clinical utility. Note: Each stage represents a minimum evidence requirement before moving to the next step. Stop/go gates indicate key questions that must be answered before progression. Failure at any stage should pause translational advancement until the relevant evidence gap is resolved. Abbreviations: AUC, area under the receiver operating characteristic curve. Created in BioRender. Asanova, A. (2026) https://BioRender.com/hn5gku1 (accessed on 17 June 2026).

For urinary biomarkers to influence oncology practice, association with cancer is not sufficient. Translation requires an evidence chain that connects specimen handling, assay performance, biological meaning, decision-specific accuracy, and patient-level benefit. Longitudinal molecular monitoring studies also illustrate that clinically meaningful interpretation depends on the relationship between biomarker dynamics and recurrence or treatment state, including emerging evidence from individualized circulating tumor DNA monitoring in UTUC [158]. At the analytical level, urinary EV assays further highlight the need for standardized isolation, recovery, normalization, and reporting before multicenter implementation [159]. In this review, we define four practical requirements for clinical translation: analytical validity, biological validity, clinical validity, and clinical utility. These domains should be evaluated sequentially, because failure at any stage limits the credibility of downstream claims (Table 3; Figure 3).

### 6.1. Analytical Validity Begins with the Specimen

Urine is a biologically variable specimen, not a standardized fluid. Whole urine, sediment, supernatant, EV fractions, selective upper-tract urine, and prostate-enriched first-catch urine contain different combinations of cells and soluble analytes. Collection after DRE, cystoscopy, ureteroscopy, TURBT, BCG instillation, biopsy, surgery, or systemic therapy can change cellular shedding, hematuria, inflammation, and nucleic-acid abundance [31,160]. Therefore, the specimen fraction, collection protocol, and sampling time must be fixed before clinical validation.

Processing delay, centrifugation, preservative use, storage temperature, freeze–thaw exposure, urine concentration, infection, hematuria, renal function, and treatment timing should be reported as potential modifiers of assay performance [168,169]. Normalization must also be analyte specific: creatinine, specific gravity, osmolality, cell number, housekeeping RNA, and spike-in controls are not interchangeable and may themselves be affected by kidney function or inflammation [170].

Analytical validation should precede outcome testing. Accuracy, repeatability, reproducibility, linearity, detection limits, interference, stability, batch effects, and computational workflow stability must be characterized using clinically realistic urine specimens and, where possible, interlaboratory testing. Technically strong performance has been reported for selected methylation and multianalyte RNA assays, but interlaboratory validation remains uncommon, particularly for EV and sequencing-based platforms [161,162].

### 6.2. Biological Validity Requires Source Attribution

Biological validity asks whether the urinary signal represents the tumor or biological state it is claimed to measure. A urinary marker associated with cancer in a case–control study should not automatically be described as tumor derived. Stronger evidence comes from paired tumor–urine concordance, comparison with adjacent nonmalignant tissue, paired plasma analysis, serial decline after treatment, cell-of-origin evidence, and re-emergence with confirmed recurrence [163,164,165].

This requirement differs across cancers. In bladder cancer, direct luminal shedding makes tumor-informed urine assays biologically plausible, but field effects and post-treatment injury still complicate interpretation. In UTUC, selective upper-tract urine strengthens anatomical attribution but does not remove procedure-related confounding. In prostate cancer, urine is prostate enriched rather than tumor specific. In RCC, urinary signals may reflect malignant tissue, benign renal epithelium, tubular injury, filtration, hematuria, or systemic biology. Source attribution is therefore essential before a urinary signal is used to support treatment escalation, de-escalation, or molecular residual disease claims.

### 6.3. Clinical Validity Must Be Decision Specific

Clinical validity should be defined by the intended decision, not by cancer association alone. Bladder surveillance assays should prioritize high-grade recurrence, progression, and BCG failure rather than any recurrence. UTUC assays should address high-grade or invasive disease, anatomical attribution, and ipsilateral recurrence after kidney-sparing treatment. Prostate assays should focus on GG2-or-higher disease and report both biopsies avoided and significant cancers missed. RCC studies should compare malignant and benign renal masses rather than cancer cases and healthy controls.

A high AUC is insufficient for clinical translation. Performance should be evaluated at prespecified thresholds using sensitivity, specificity, predictive values, calibration, and confidence intervals. The assay should then be tested against contemporary standards of care, including cystoscopy and cytology, ureteroscopy and imaging, PSA density and MRI, or renal-mass imaging. Incremental value requires evidence of improved discrimination, calibration, decision-curve net benefit, or clinically meaningful reclassification beyond existing pathways [166].

### 6.4. Clinical Utility and Positioning of Clinically Available Urinary Biomarker Assays

Clinical utility requires more than diagnostic accuracy. A biomarker should improve the balance between benefit and harm when acted upon, with relevant outcomes including invasive procedures avoided, significant cancers missed, diagnostic delay, treatment-failure detection, complications, quality of life, adherence, and cost-effectiveness [166]. Procedure avoidance alone is therefore insufficient without corresponding evidence of oncological safety.

Commercial availability, regulatory authorization, guideline inclusion, and demonstrated clinical utility represent distinct levels of translational maturity [166,167]. This distinction is evident in bladder cancer, where several urinary molecular assays show favorable diagnostic or surveillance performance but remain largely adjunctive to cystoscopy and risk-adapted assessment [66,160]. In prostate cancer, urinary RNA- and extracellular-vesicle-based assays are more established as pre-biopsy decision-support tools, with value depending on the tested population and incremental information beyond PSA, MRI, and clinical risk factors [96,99,110,114].

Real-world adoption also depends on reproducibility, laboratory complexity, turnaround time, reimbursement, reporting, regulatory requirements, confirmatory testing, and subgroup performance. Table 4 therefore summarizes selected clinically available urinary biomarker assays according to intended use, regulatory or commercial status, major strengths and limitations, and current guideline positioning [6]. Regulatory authorization or commercial availability should not be interpreted as evidence that an assay can independently replace cystoscopy, ureteroscopy, MRI, biopsy, or histopathological assessment.

Current guideline positioning remains more conservative than the expanding biomarker literature. In NMIBC, the 2026 EAU guideline states that urinary molecular markers have not been accepted for routine primary diagnosis and cannot currently replace cystoscopy, although recent randomized evidence supports marker-guided surveillance strategies in selected follow-up settings; the current AUA/SUO guideline similarly assigns urinary biomarkers a limited adjunctive role, including UroVysion FISH for assessment of BCG response and adjudication of equivocal cytology rather than routine replacement of cystoscopic surveillance [171,172]. In UTUC, the 2025/2026 EAU guideline recommends voided urinary cytology in suspected disease and recognizes molecular urinary assays as potentially useful adjuncts, but CT urography, ureteroscopy, biopsy, and selective cytology remain the principal diagnostic standards; the AUA/SUO guideline likewise centers diagnosis on imaging, ureteroscopy/biopsy, and upper-tract cytologic washing rather than molecular urine testing [92,173,174]. In prostate cancer, guideline positions are more permissive but remain selective: the 2026 AUA/SUO early-detection framework allows adjunctive urine or serum biomarkers when additional risk stratification would materially influence the biopsy decision, whereas the 2026 EAU guideline concludes that no individual urine test can yet be recommended for routine clinical practice and continues to regard MPS/ExoDx as investigational in its diagnostic framework [175,176,177]. Current NCCN early-detection guidance similarly incorporates selected urine-based assays into pre-biopsy risk refinement rather than positioning them as stand-alone screening or diagnostic tests. In RCC, contemporary EAU guidance remains imaging- and pathology-centered; urinalysis is part of routine evaluation and urinary cytology may be considered for central renal masses to exclude urothelial carcinoma, but no urinary molecular biomarker is recommended for routine RCC detection, renal-mass characterization, or surveillance [178]. Overall, current guidelines support the central translational message of this review: urinary biomarkers are increasingly recognized as decision-support tools in selected clinical pathways, but evidence remains insufficient for broad substitution of established imaging, endoscopic, or histopathological standards.

### 6.5. Real-World Implementation and Health-System Accessibility

Clinical translation requires more than diagnostic accuracy. A urinary biomarker must retain reproducibility across sites, workflows, operators, and patient populations. This is particularly important for urine, where collection timing, hydration, preservative use, processing delay, storage conditions, centrifugation, normalization, and the analyzed fraction can influence biomarker recovery and assay performance [31]. These challenges are amplified for extracellular-vesicle and multi-omics approaches, in which isolation methods, recovery efficiency, contamination, and normalization contribute additional inter-study variability [159,179]. Accordingly, translational frameworks for liquid-biopsy biomarkers increasingly emphasize standardized pre-analytical workflows, predefined quality-control criteria, reproducible targeted assays, independent validation, and multicenter testing before clinical implementation [160,180].

Implementation must also demonstrate economic value within the intended clinical pathway rather than simply an acceptable assay price. The relevant question is whether biomarker-guided testing can safely reduce downstream cystoscopy, ureteroscopy, imaging, or biopsy while preserving detection of clinically significant disease. In hematuria evaluation, the cost-effectiveness of urinary testing has been shown to depend strongly on test price, cancer prevalence, and downstream procedure use [181], while a later appraisal of bladder-cancer economic models identified substantial uncertainty and inconsistent conclusions regarding urinary biomarkers [182]. Similar considerations apply in prostate cancer: integration of SelectMDx with MRI has been modelled to produce modest quality-adjusted life-year (QALY) gains and cost savings, but these estimates depend on assumptions regarding MRI utilization, biopsy avoidance, and missed clinically significant cancers [183]. Procedure avoidance should therefore always be interpreted alongside its clinical consequences, particularly the number and grade of cancers missed [114].

Real-world utility further depends on whether an assay can be delivered within available health-system infrastructure. Sequencing, methylation, EV, and high-dimensional multi-omics assays may require specialized platforms, trained personnel, bioinformatics support, stringent quality assurance, reliable specimen transport, and sustainable reimbursement. These requirements may limit accessibility in settings with restricted molecular-diagnostic capacity. Conversely, standardized automated or point-of-care assays may provide greater system-level value when they safely reduce dependence on specialist procedures or scarce imaging resources. Recent randomized NMIBC trials provide an important proof of principle that biomarker-guided surveillance can reduce cystoscopy exposure in selected patients, although broader oncological safety, resource implications, and cost-effectiveness remain context dependent [184,185]. Implementation studies should therefore evaluate assay failure rates, turnaround time, inter-laboratory reproducibility, resource requirements, downstream procedure use, patient-relevant outcomes, and cost-effectiveness alongside conventional measures of diagnostic performance. Ultimately, clinical maturity requires not only analytical and clinical validity, but reproducible net clinical benefit within the infrastructure, resources, and referral pathways of the health system in which the biomarker is intended to be used.

## 7. Designing the Next Generation of Urinary Biomarker Studies

Future urinary biomarker studies should move beyond retrospective, single-center case–control comparisons toward prospective designs that test whether biomarkers improve clinical decisions. Larger cohorts alone will not correct the main limitations of the field: uncertain source attribution, sparse longitudinal sampling, inconsistent specimen processing, anatomical ambiguity, overfitted models, discordant results, and limited evidence that biomarker-guided management is safe. The priority for clinical oncology is therefore not only to show that a urinary signal is associated with cancer, but to show that acting on the result can reduce unnecessary procedures, identify clinically significant disease, or detect treatment failure without unacceptable harm (Table 5).

### 7.1. Paired Tissue–Urine Validation

Paired tissue–urine studies should form the biological foundation of urinary biomarker translation. A minimum design should collect the relevant urine fraction, tumor tissue, adjacent nonmalignant tissue, plasma, and the clinical reference specimen from the same patient. Tumor–urine concordance can establish whether urinary mutations, methylation patterns, or expression programs derive from the malignant clone, whereas adjacent tissue and paired plasma help distinguish field alteration, benign tissue release, local urinary enrichment, and systemic tumor products [163,186]. The primary endpoint should be source attribution and compartment-specific concordance, not cancer-versus-control discrimination alone.

### 7.2. Longitudinal Monitoring Studies

Cross-sectional studies cannot distinguish persistent disease from acute procedural release. Sampling should include pretreatment baseline, immediate perturbation, recovery, surveillance, and recurrence windows after TURBT, BCG, ureteroscopy, kidney-sparing UTUC treatment, prostate manipulation or biopsy, nephrectomy, or systemic therapy. Serial bladder-cancer studies have shown that postoperative urinary methylation and tumor-DNA trajectories can provide recurrence information beyond a single baseline measurement [151,164]. Required outputs include analyte-specific clearance curves, within-patient trajectories, molecular lead time, and time-dependent prediction models. Two-point pre/post comparisons should be avoided because they cannot reliably separate transient release, true clearance, persistence, and recurrence.

### 7.3. Cross-Fraction and Anatomical Localization Studies

Sediment, cell-free supernatant, and EV fractions are biologically non-equivalent and should be compared from the same void. Comparative methylation studies have shown that the optimal fraction differs by marker, with some targets performing best in sediment and others in supernatant or whole urine [187]. EV-based studies similarly demonstrate information not captured by bulk urine alone [28,188]. Studies should therefore report yield, failure rate, reproducibility, complementarity, and incremental clinical value for each fraction.

Anatomical localization should be evaluated separately from cancer detection. UTUC studies should compare pre-instrumentation voided urine, bladder urine, ipsilateral selective upper-tract urine, contralateral urine where ethically justified, and matched tumor tissue. Selective upper-tract cytology and FISH can improve sensitivity relative to voided sampling, but molecular localization remains insufficiently validated [76,88]. Tumor-informed sequencing should determine whether detected clones are lesion specific, shared across sites, or compatible with a pan-urothelial field. This is essential because voided positivity alone should not be assumed to identify the anatomical site of disease.

### 7.4. Artificial Intelligence and Biology-Informed Multimodal Prediction

Future urinary biomarker models may derive greater clinical value from integration with complementary imaging, clinical, and genomic information than from increasingly complex urine signatures alone. Evidence is most developed in prostate cancer, where combining urinary ExoDx-, SelectMDx-, or MyProstateScore-derived information with PSA-based variables and multiparametric magnetic resonance imaging (mpMRI)/PI-RADS can improve risk stratification and refine the trade-off between biopsies avoided and clinically significant cancers missed [110,112,113,114,189].

However, the incremental value of urinary biomarkers beyond contemporary MRI-based risk assessment remains uncertain and appears to be context dependent. In biopsy-naive men, prospective evidence indicates that an MRI-first strategy may provide greater clinical utility than SelectMDx-based or biomarker-first sequential strategies, whereas potential complementary value appears more plausible in patients with residual diagnostic uncertainty, particularly those with equivocal PI-RADS 3 findings or negative/low-suspicion MRI and persistent clinical suspicion [114,190,191,192]. Urinary biomarkers should therefore not currently be considered routine additions to PI-RADS assessment; their more plausible role is as selective second-line risk-refinement tools when MRI and established clinical variables leave uncertainty. Future multimodal studies should demonstrate incremental discrimination, calibration, decision-curve net benefit, biopsy reduction, and the proportion of clinically significant cancers missed beyond models incorporating PI-RADS and established clinical predictors.

In bladder cancer, machine-learning integration of urinary exosomal miRNAs with clinical, demographic, and routine laboratory variables has similarly improved discrimination over either data domain alone, providing proof-of-concept for multimodal urinary modelling [193]. Comparable urine-centered evidence remains sparse in UTUC and RCC. Artificial intelligence (AI) may extend these approaches by integrating urinary molecular signals with imaging, clinical variables, genomic information, and longitudinal measurements, thereby combining complementary information on tumor biology, anatomical localization, baseline risk, and disease dynamics [194].

Greater algorithmic complexity, however, does not establish clinical utility. AI-enabled multimodal models should be biology-informed and demonstrate incremental value beyond established clinical and imaging models, while addressing overfitting, data leakage, missing modalities, inter-platform variability, and dataset shift [195]. Model development, preprocessing, feature selection, and decision thresholds should be prespecified. Independent evaluation should assess discrimination, calibration, decision-curve analysis, and clinically relevant benefit–harm endpoints rather than AUC alone [166,167,192,196,197,198]. Ultimately, multicenter external validation and prospective clinical-impact studies will be required to determine whether multimodal prediction improves clinical decisions rather than predictive performance alone.

### 7.5. Prospective Decision-Impact Studies

The decisive evidence will come from prospective studies in which biomarker results change management. These studies should enroll the intended-use population, apply a prespecified testing algorithm, define confirmatory procedures, and measure both benefit and harm. Testing thresholds and analytical procedures should be specified before evaluation, and performance should include calibration and decision-curve analysis in addition to discrimination [199,200]. In bladder cancer, the key question is whether urinary biomarkers can safely reduce cystoscopy burden without missing HG recurrence. In prostate cancer, urine-plus-MRI or urine-after-equivocal-MRI pathways should report both biopsies avoided and GG ≥ 2 cancers missed. In UTUC, studies should determine whether biomarkers can prioritize ureteroscopy or support surveillance after kidney-sparing treatment. In RCC, urinary assays should remain focused on renal-mass characterization or exploratory treatment monitoring until source attribution improves.

Biomarker-guided management trials should use clinically meaningful endpoints, including significant disease missed, procedures avoided, diagnostic delay, complications, patient-reported outcomes, and cost. Randomized bladder-cancer trials have begun to show that biomarker-guided surveillance can reduce cystoscopy burden, although long-term oncological safety remains essential [184,185]. Comparable studies are needed for UTUC surveillance after kidney-sparing treatment, urine-plus-MRI biopsy pathways in prostate cancer, and paired urine–plasma monitoring after nephrectomy. Analogous lessons from pediatric neuro-oncology and cerebrospinal fluid-based liquid biopsy reinforce that translation requires longitudinal response assessment, compartment-specific interpretation, and evidence that molecular information can support clinically meaningful decisions [201,202].

### 7.6. Discordance and Negative-Result Studies

Future studies should deliberately analyze failures, not only concordant positive cases. Disease-positive but urine-negative patients can reveal anatomical non-access, poor shedding, fraction mismatch, analyte degradation, assay-target mismatch, or inadequate sensitivity. Biomarker-positive but reference-negative states should be followed longitudinally to determine whether they represent molecular anticipation, field alteration, inflammation, injury, anatomical misattribution, or analytical false positivity. Reporting should include reason-specific false-negative rates and the eventual outcomes of discordant cases.

## 8. Conclusions

The central contribution of this review is a shift from platform-centered classification toward causal and decision-oriented interpretation of urinary biomarkers. Rather than assuming that analytical detection is equivalent to tumor detection, the proposed framework links each urinary signal to its biological source, anatomical route into urine, specimen fraction, temporal and perturbational context, and intended clinical decision. This approach provides a common interpretive structure across bladder cancer, UTUC, prostate cancer, and RCC despite their markedly different relationships with the urinary compartment.

Within this framework, urinary biomarkers should be interpreted as decision-support tools rather than as direct surrogates of tumor presence. Their clinical meaning depends on the anatomical route into urine, the biological source of the signal, the analyzed specimen fraction, sampling time, and the intended oncological decision. These factors differ substantially across urological cancers. Bladder cancer has the strongest anatomical access and the most mature evidence base; UTUC requires careful distinction between voided and selective urine because localization is the central challenge; prostate urine assays mainly support biopsy triage and active-surveillance refinement; and RCC urinary biomarkers remain exploratory because source attribution is unresolved.

The practical implication is that urinary biomarkers should be validated according to the decision they are expected to change. A test used for hematuria evaluation, cystoscopy deferral, UTUC localization, prostate biopsy triage, renal-mass characterization, molecular residual disease assessment, or treatment monitoring requires different thresholds, endpoints, and acceptable harms. Positive results require source attribution and clinical actionability, whereas negative results justify de-escalation only when anatomical access, shedding, fraction suitability, and analytical recovery are sufficient.

The next phase of urinary biomarker research should therefore prioritize prospective decision-impact studies, paired tissue–urine validation, longitudinal monitoring, discordance adjudication, and comparison with contemporary clinical pathways. Clinical maturity should be defined not by biomarker association or area under the curve alone, but by evidence that biomarker-guided care reduces unnecessary procedures, detects clinically significant disease, avoids unacceptable missed cancers, and improves patient management.

## Figures and Tables

**Table 1:** Specimen fractions as clinically distinct urinary sampling compartments.

Specimen Compartment	Enriched Signal	Best Clinical Use	Main Clinical Limitation
Whole voided urine	Mixed urinary-tract cells, soluble molecules, debris, vesicles, microbes, and cell-free nucleic acids	Multiplex testing, cytology, hematuria evaluation, and bladder-cancer triage	Limited source attribution because signals may originate from bladder, upper tract, kidney, urethra, prostate, inflammation, or blood
First-catch urine	Urethral and prostate-enriched material	Prostate biomarker testing, especially RNA- or EV-based biopsy-triage assays	Sensitive to collection volume, prior voiding, urethral contamination, and variable prostate contribution
Post-DRE urine	Mechanically enriched prostate cells, secretions, RNA, proteins, and vesicles	PCA3, HOXC6/DLX1, and related prostate RNA panels	Operator variation and incomplete tumor specificity because benign prostate tissue also contributes signal
Sediment or cell pellet	Tumor and benign epithelial cells, leukocytes, erythrocytes, bacteria, and cell-associated nucleic acids	Cytology, mutation, methylation, and cellular RNA assays	Variable cell yield, blood contamination, lysis, and centrifugation effects
Supernatant or cfDNA-enriched supernatant	Soluble proteins, metabolites, cytokines, extracellular nucleic acids, and residual vesicles	Proteomics, metabolomics, mutation testing, and methylation testing	Dilution, enzymatic degradation, genomic-DNA contamination, and uncertain cellular origin
Extracellular-vesicle fraction	Vesicle-protected RNA, DNA, proteins, lipids, and surface markers from mixed cell sources	Multi-omic assays and cell-of-origin enrichment strategies	Isolation bias, non-tumor vesicle abundance, poor comparability across methods, and unresolved normalization
Selective upper-tract urine	Ipsilateral renal pelvic or ureteral cells and molecular material	UTUC localization, cytology, and molecular profiling	Instrumentation-related injury, hematuria, low volume, saline dilution, and limited generalizability to voided urine
Catheterized or diversion urine	Bladder-focused urine or urine mixed with bowel-derived material, mucus, colonizing microbes, and inflammatory products	Selected surveillance settings and patients unable to void	Mechanical injury, infection, mucus, chronic inflammation, colonization, and altered diversion anatomy

Abbreviations: cfDNA, cell-free DNA; DRE, digital rectal examination; EV, extracellular vesicle; UTUC, upper-tract urothelial carcinoma.

**Table 2:** Clinical decision contexts that define the interpretation and validation of urinary biomarkers across urological cancers.

Clinical Decision	Cancer Setting	Test Role	Harm to Avoid	References
Hematuria evaluation	Bladder cancer; UTUC	Rule-out or enrichment before cystoscopy/imaging	Missed high-grade urothelial carcinoma or delayed diagnosis	[21,34,38]
Cystoscopy deferral or prioritization	Bladder cancer surveillance	Triage, surveillance support, or risk stratification	False reassurance, delayed recurrence detection, unnecessary cystoscopy	[5,6,21,34]
Upper-tract localization	UTUC; patients with prior or synchronous bladder cancer	Anatomical localization and selection for ureteroscopy/selective sampling	Misattribution of bladder versus upper-tract disease	[17,24,33]
Prostate biopsy triage	Prostate cancer	Enrichment for clinically significant disease before biopsy	Missed Grade Group (GG) ≥ 2 disease or unnecessary biopsy	[39,40,41,42,43]
Active-surveillance refinement	Prostate cancer; selected bladder cancer contexts	Monitoring and escalation support	Delayed detection of progression or overtreatment of stable disease	[14,18,19,36]
Molecular residual disease assessment	Bladder cancer; UTUC; selected RCC contexts	Detection of persistent tumor-derived material after curative-intent treatment	Misclassification of field effect, injury, or transient post-treatment release as residual disease	[15,35,36]
Treatment-response monitoring	Bladder cancer treated with BCG; systemic therapy settings; RCC	Pharmacodynamic assessment, response monitoring, or escalation support	Confusing immune activation or treatment injury with tumor clearance	[29,36,37]
Renal-mass characterization	RCC	Adjunctive discrimination of malignant versus benign renal lesions	Overdiagnosis, unnecessary intervention, or false reassurance in malignant disease	[7,8,20]

Abbreviations: BCG, Bacillus Calmette–Guérin; GG, Grade Group; RCC, renal cell carcinoma; UTUC, upper-tract urothelial carcinoma.

**Table 3:** Minimum evidence standards for clinical translation of urinary biomarkers.

Evidentiary Domain	Central Question	Minimum Evidence	Preferred Study Design	Decision-Relevant Endpoint	Common Failure Mode
Pre-analytical validity	Does the collected specimen reproducibly represent the intended urinary compartment?	Prespecified urine fraction, collection route, volume, timing relative to manipulation or treatment, processing interval, centrifugation, stabilizer, storage, freeze–thaw exposure, hematuria, infection, renal function, and concentration adjustment [31,160]	Prospective method-comparison and stability studies using clinically representative samples	Analyte recovery, failure rate, within-person variability, stability under transport and storage conditions	Unreported specimen fraction; inconsistent handling between cases and controls; post-procedural samples pooled with baseline samples
Analytical validity	Does the assay measure the target accurately and reproducibly across its claimed range?	Accuracy, precision, linearity, limit of detection, limit of quantification where relevant, interference, stability, batch control, normalization, and interlaboratory reproducibility [161,162]	Blinded analytical validation using reference materials, contrived low-positive samples, and clinical specimens across laboratories	Bias, coefficient of variation, detection probability, invalid-result rate, interlaboratory concordance	Limit of detection inferred from patient data; no interference testing; computational pipeline altered after validation
Biological validity and source attribution	Does the urinary signal arise from the tumor or biological state it is claimed to represent?	Paired tumor–urine concordance; comparison with adjacent nonmalignant tissue and plasma; serial decline after treatment; cell-of-origin or clonal evidence; exclusion of inflammation, field change, and renal injury [163,164,165]	Prospective paired tissue, urine, plasma, and serial post-treatment study using tumor-informed or source-resolved assays	Clonal concordance, source-specific signal, postoperative clearance, re-emergence with recurrence	Cancer association assumed to establish tumor derivation; no relevant benign or inflammatory comparators
Temporal calibration	When is the result interpretable relative to treatment, instrumentation, and disease evolution?	Prespecified baseline, acute perturbation, clearance, surveillance, and recurrence windows	Longitudinal cohort with fixed sampling intervals and treatment-specific time points	Clearance kinetics, within-person trajectory, molecular lead time, reproducibility of change	Immediate postoperative positivity labeled residual disease; heterogeneous sampling intervals
Clinical validity	Does the assay predict the intended disease-specific endpoint in the intended-use population?	Prespecified threshold, clinically meaningful endpoint, blinded assessment, representative disease spectrum, and external validation	Prospective multicenter cohort in the target clinical population	High-grade recurrence for bladder cancer; invasive or high-grade UTUC; GG2 or higher prostate cancer; malignant versus benign renal mass or post-nephrectomy recurrence	Cancer-versus-healthy-control design; post hoc threshold selection; reporting AUC without sensitivity, specificity, predictive values, and confidence intervals
Incremental value	Does the biomarker improve prediction or decision-making beyond standard care?	Direct comparison with contemporary clinical models and procedures, with assessment of calibration and net benefit [166]	Prospective head-to-head study incorporating the biomarker into the existing clinical pathway	Change in discrimination, calibration, decision-curve net benefit, and clinically meaningful reclassification	Comparison with no testing or an obsolete comparator; statistically significant AUC increase without clinical net benefit
Clinical utility	Does biomarker-guided care improve the balance of benefits and harms?	Demonstrated management change without unacceptable missed significant disease; assessment of complications, diagnostic delay, patient-reported outcomes, adherence, and costs [167]	Randomized biomarker-strategy trial, prospective impact study, or comparative-effectiveness design	Procedures avoided, significant cancers missed, time to diagnosis, earlier treatment-failure detection, complications, quality of life, and cost-effectiveness	Diagnostic accuracy presented as evidence of utility; procedures avoided reported without missed-cancer outcomes
Implementation and equity	Can the assay be deployed reliably, affordably, and equitably across real-world settings?	Defined laboratory requirements, turnaround time, regulatory pathway, reporting format, subgroup performance, and external validation across ancestry, geography, infection burden, and renal-function strata	Multisite implementation study with post-deployment performance monitoring	Turnaround time, invalid-result rate, laboratory concordance, calibration across settings, access, uptake, and subgroup performance	Validation restricted to specialized centers and predominantly European-ancestry cohorts; no assessment of infection, CKD, or resource constraints

Abbreviations: AUC, area under the receiver operating characteristic curve; CKD, chronic kidney disease; GG, Grade Group; UTUC, upper-tract urothelial carcinoma.

**Table 4:** Clinical positioning of selected commercially available urinary biomarker assays.

Assay	Biomarker/Specimen	Main Clinical Use	Regulatory/Commercial Status *	Key Strengths/Limitations and Guideline Position
Bladder cancer				
UroVysion FISH [21,62,66]	Aneuploidy/9p21 loss; urinary cells	Diagnosis and NMIBC surveillance adjunct	FDA-approved	Established cellular assay; requires adequate cellularity and does not localize disease. Adjunctive; does not replace cystoscopy.
NMP22 BladderChek [21,62,63]	NMP22 protein; voided urine	Hematuria/detection adjunct	FDA-cleared	Rapid point-of-care test, but specificity is reduced by hematuria, infection and inflammation. Not a stand-alone replacement for cystoscopy.
Bladder EpiCheck [66,71]	DNA methylation; urinary cells	NMIBC surveillance	FDA-cleared; commercially available	High NPV, especially for HG recurrence; lower sensitivity for LG disease. Emerging surveillance adjunct.
Xpert Bladder Cancer Monitor [66]	5-mRNA panel; voided urine	NMIBC surveillance	Commercially available	Automated assay with high NPV for HG recurrence; evidence remains insufficient for routine cystoscopy replacement. Adjunctive.
Cxbladder [66,162]	5-mRNA classifier; voided urine	Hematuria evaluation/surveillance	Commercially available	Strong rule-out performance in selected pathways; utility is population- and pathway-dependent. Selective adjunctive use.
Prostate cancer				
Progensa PCA3 [18,103,108]	PCA3/PSA RNA; post-DRE urine	Repeat-biopsy risk assessment	FDA-approved	Established prostate-enriched urinary RNA test, but limited discrimination of clinically significant disease. Selective role.
ExoDx Prostate (EPI) [39,96,110]	Exosomal RNA; first-catch urine	Pre-biopsy GG ≥ 2 risk stratification	Commercial laboratory test	No DRE required; validated for clinically significant disease, but does not localize lesions. Biopsy decision-support tool.
SelectMDx [40,99,114]	HOXC6/DLX1 mRNA; post-DRE urine	Pre-biopsy risk stratification	Commercially available	May reduce unnecessary biopsy; optimal integration with MRI remains uncertain. Selective adjunct.
MPS2 [97,101]	18-gene urinary expression model	Pre-biopsy GG ≥ 2 risk stratification	Commercial laboratory test	Focused on clinically significant disease; pathway-level clinical utility and MRI integration require further validation. Emerging decision-support test.

* Regulatory status and commercial availability are jurisdiction-specific and do not imply that an assay can replace standard imaging, endoscopy, biopsy, or histopathology. Abbreviations: DRE, digital rectal examination; FDA, U.S. Food and Drug Administration; FISH, fluorescence in situ hybridization; GG, Grade Group; HG, high-grade; LG, low-grade; MRI, magnetic resonance imaging; NMIBC, non-muscle-invasive bladder cancer; NPV, negative predictive value; PCA3, prostate cancer antigen 3.

**Table 5:** Priority designs for next-generation urinary biomarker studies.

Study Architecture	Core Specimens or Sampling	Primary Biological Question	Preferred Endpoint	Principal Failure to Avoid
Paired multi-compartment study	Urine fractions, tumor, adjacent tissue, plasma, immune cells	Where does the urinary signal originate?	Tumor–urine clonal or molecular concordance	Equating cancer association with tumor derivation
Dense longitudinal study	Serial samples before and after treatment or instrumentation	Does the signal represent perturbation, clearance, persistence, or recurrence?	Analyte-specific trajectory and recurrence-linked persistence	Two-point pre/post comparisons
Cross-fraction study	Sediment, supernatant, and EVs from the same void	Which fraction contains complementary clinical information?	Incremental value and reproducibility by fraction	Comparing unmatched fractions or samples
Anatomical localization study	Voided, bladder, and selective upper-tract urine with matched tissue	Can the assay localize the lesion or only detect urothelial abnormality?	Site- and side-specific accuracy	Assuming voided positivity identifies disease location
Biology-informed multimodal model	Malignant, field, immune, contextual, imaging, and clinical modules	Which biological modules add nonredundant information?	Calibration, net benefit, and external validation	Opaque high-dimensional overfitting
Biomarker-guided management trial	Intended-use clinical population with a prespecified testing algorithm	Does biomarker-guided care improve the benefit–harm balance?	Significant disease missed and procedures avoided	Using AUC as the primary clinical endpoint
Negative-result adjudication study	Disease-positive but urine-negative patients; matched urine fractions, tumor tissue, adjacent tissue when available, cytology, imaging, endoscopy, and follow-up	Why was cancer missed: anatomical non-access, poor shedding, fraction mismatch, analyte degradation, assay-target mismatch, or inadequate sensitivity?	Reason-specific false-negative rate and outcome of biomarker-positive/reference-negative states	Treating all negative results as equivalent or selectively reporting only concordant cases

Abbreviations: AUC, area under the receiver operating characteristic curve; EV, extracellular vesicle.

## Data Availability

No new data were created or analyzed in this study. Data sharing is not applicable to this article.

## Supplementary Materials

The following supporting information can be downloaded at https://www.mdpi.com/article/10.3390/diagnostics16172810/s1. Figure S1: Biological state, temporal trajectory and urinary observability in longitudinal biomarker interpretation; Table S1: Biological signal modules represented in urine across urological cancers; Table S2: Cross-cancer biological states represented by urinary biomarkers; Table S3: Framework for interpreting discordance between urinary biomarkers and conventional assessment.

## Abbreviations

The following abbreviations are used in this manuscript:

AI	Artificial intelligence
AUC	Area under the receiver operating characteristic curve
BC	Bladder cancer
BCG	Bacillus Calmette–Guérin
BPH	Benign prostatic hyperplasia
BTA	Bladder tumor antigen
cfDNA	Cell-free DNA
CIS	Carcinoma in situ
CKD	Chronic kidney disease
CNA	Copy-number alteration
ctDNA	Circulating tumor DNA
DRE	Digital rectal examination
EVs	Extracellular vesicles
FDA	U.S. Food and Drug Administration
FISH	Fluorescence in situ hybridization
GG	Grade Group
HG	High-grade
LG	Low-grade
MMP	Matrix metalloproteinase
mpMRI	Multiparametric magnetic resonance imaging
MPS	Michigan Prostate Score/MyProstateScore
MPS2	MyProstateScore 2.0
MRI	Magnetic resonance imaging
MRD	Molecular residual disease
NMIBC	Non-muscle-invasive bladder cancer
NPV	Negative predictive value
PC	Prostate cancer
PCA3	Prostate cancer antigen 3
PI-RADS	Prostate Imaging Reporting and Data System
PSA	Prostate-specific antigen
QALY	Quality-adjusted life year
RCC	Renal cell carcinoma
SNV	Single-nucleotide variant
TDM	Treatment-decision monitoring
TURBT	Transurethral resection of bladder tumor
UTI	Urinary tract infection
UTUC	Upper-tract urothelial carcinoma
VHL	von Hippel–Lindau
