# Supplementary material for: Urinary Biomarkers in Urological Oncology: From Biological Origin to Clinical Decision-Making—A Narrative Review
Source: Diagnostics (Basel). 2026 Sep 1;16(17):2810. doi: 10.3390/diagnostics16172810 (PMC13565365; doi:10.3390/diagnostics16172810)
Supplement: Supplementary file 1 [file diagnostics-16-02810-s001.zip › diagnostics-4461029-supplementary.pdf]

Supplementary Materials

Suppl. Table S1. Biological signal modules represented in urine across urological cancers.

Biological signal module	Principal sources and representative signals	Evidence across urological cancers	Potential biological meaning	Major alternative explanations	Evidence maturity
Malignant burden and molecular identity [8,17,19-21,44]	Malignant cells and tumor-derived material; somatic mutations, CNAs, structural variants, tumor-associated methylation, cancer-linked RNA, malignant cytology and tumor-associated EV cargo	**Bladder:** direct luminal shedding supports extensive validation of cytology, mutation, methylation and RNA assays. **UTUC:** direct but upstream shedding is detectable, although voided-urine sensitivity and localization are limited by dilution, intermittent shedding and obstruction. **Prostate:** RNA and EV assays are clinically developed, but signals depend on first-catch or post-DRE enrichment rather than direct tumor contact. **RCC:** urinary DNA, RNA, proteins and EVs are detectable, but tumor fraction is often low and attribution to malignant cells remains uncertain	Malignant-cell presence, clonal identity, molecular subtype, grade, treatment response or residual disease	Field-derived mutations, absence of the targeted alteration, low shedding, dilution, genomic DNA contamination and non-tumor EVs	Established for cytology and selected bladder/prostate assays; emerging for urinary MRD, UTUC and RCC
Field alteration, epithelial injury and regeneration [12,13,23, 45-47]	Molecularly altered but nonmalignant urothelium; injured urothelial, prostate or renal epithelial cells; mutations, methylation, stress-response RNA, injury proteins and cellular debris	**Bladder and UTUC:** field cancerization is biologically well established across the urothelium and can contribute persistent urinary mutation, methylation and protein signals after visible tumor removal. **Prostate:** benign epithelial secretion, prostatitis and procedural injury may alter urinary transcript and protein profiles, but a defined cancerized-field signal is less established. **RCC:** tubular and	Carcinogen-exposed or recurrence-prone field, occult urothelial disease, epithelial turnover, obstruction, treatment injury or regeneration	Age-related clonal expansion, infection, calculi, instrumentation, surgery, intravesical therapy and benign renal injury	Strong biological evidence for urothelial field effects; limited clinical ability to distinguish field, injury and viable tumor

		parenchymal injury is readily represented in urine, although it is difficult to separate from tumor-associated renal effects			
Immune activation, suppression and treatment engagement [22, 48-52]	Neutrophils, macrophages, lymphocytes, dendritic cells and urothelial cells; cytokines, chemokines, immune transcripts, proteases, checkpoint-associated molecules and immune-cell EVs	**Bladder:** direct tumor–urine contact and intravesical BCG provide the strongest evidence that urinary immune cells and mediators can reflect the local tumor immune environment and treatment engagement. **UTUC:** biological plausibility exists, but upstream dilution and procedure-related inflammation limit interpretation. **Prostate:** urinary inflammation is strongly confounded by prostatitis, BPH and prostate manipulation. **RCC:** immune biology is clinically important, but correspondence between urinary immune signals and the intratumoral immune state remains insufficiently validated	Immune recruitment, innate or adaptive activation, pharmacodynamic response, immune exhaustion or myeloid suppression	UTI, cystitis, prostatitis, instrumentation, tissue injury, bystander lymphocytes and nonproductive inflammation	Moderate and rapidly developing in bladder cancer; exploratory in UTUC, prostate cancer and RCC
Stromal, vascular and matrix remodeling [12,53-55]	Fibroblasts, endothelial cells, extracellular matrix, platelets and injured tissue; VEGF, TGF-β-related factors, MMPs, TIMPs, collagen fragments and stromal or endothelial EV cargo	**Bladder:** urinary angiogenic and matrix-remodeling proteins have been associated with invasive or biologically active disease, although their cellular sources are mixed. **UTUC:** urinary evidence is limited and often extrapolated from bladder cancer or tissue studies. **Prostate:** stromal remodeling is biologically important, but validated urinary representations are sparse. **RCC:** angiogenic and renal matrix pathways are highly relevant,	Angiogenesis, invasion, hypoxia, fibroblast activation, fibrosis, immune exclusion or treatment resistance	Wound healing, renal fibrosis, proteinuria, infection, obstruction, vascular injury and postoperative repair	Mechanistically established in tumor tissue; urinary source attribution and clinical validation remain early

		particularly through VHL–HIF–VEGF biology, but urinary levels are affected by renal disease and systemic production			
Microbial, benign inflammatory and renal context [7,56-60]	Urinary bacteria, microbial products, benign prostate or urothelial cells, circulating molecules, glomeruli and renal tubules; microbial DNA, taxa, metabolites, albumin, renal EVs and injury proteins	**Bladder:** most urinary-microbiome cancer studies have focused on bladder cancer, but findings remain heterogeneous and sensitive to collection and contamination. **UTUC:** direct evidence is sparse. **Prostate:** urinary and genitourinary microbiomes are associated with prostatitis and prostate cancer, but anatomical attribution and causality remain uncertain. **RCC:** urinary composition is strongly conditioned by glomerular filtration, tubular handling, nephron mass and renal injury; microbiome evidence is limited	Microbial ecology, benign inflammation, carcinogen metabolism, systemic host response, renal filtration or tubular injury	Antibiotics, collection contamination, sex, geography, CKD, proteinuria, hydration, BPH, prostatitis, calculi and hematuria	Well established as contextual biology and a source of confounding; exploratory as a cancer classifier

Note: Evidence maturity refers to the extent of biological validation and clinical translation of the signal module, not to every individual analyte within that module. Cross-cancer descriptions are based on the consistency and depth of evidence within each disease and should not be interpreted as quantitative rankings derived from direct head-to-head studies. **Abbreviation:** BCG, Bacillus Calmette–Guérin; BPH, benign prostatic hyperplasia; cfDNA, cell-free DNA; CKD, chronic kidney disease; CNA, copy-number alteration; DRE, digital rectal examination; EV, extracellular vesicle; HIF, hypoxia-inducible factor; MMP, matrix metalloproteinase; MRD, molecular residual disease; RCC, renal cell carcinoma; TGF-β, transforming growth factor-β; TIMP, tissue inhibitor of metalloproteinases; UTI, urinary tract infection; UTUC, upper-tract urothelial carcinoma; VEGF, vascular endothelial growth factor; VHL, von Hippel–Lindau.

Suppl. Table S2. Cross-cancer biological states represented by urinary biomarkers.

Biological state	Urinary pattern	Bladder cancer	UTUC	Prostate cancer	RCC	Strongest inference	Principal limitation
**Residual malignant burden**	Persistent or re-emerging tumor-informed signal after treatment	Persistent urinary tumor DNA after TURBT or intravesical therapy has been associated with residual disease, recurrence, and longitudinal disease status [36,67,143]. Persistent malignant cytology provides complementary cellular evidence but is less molecularly specific.	Tumor-matched DNA in ipsilateral selective urine after kidney-sparing treatment would provide the strongest evidence of residual upper-tract disease. However, prospective evidence linking post-ablation urinary persistence to recurrence remains limited; surveillance still depends on ureteroscopy, cytology, and imaging [91,144].	A high-risk urinary RNA score after a negative or low-grade biopsy indicates an increased probability of unsampled GG2 or higher disease rather than postoperative residual disease [101].	Tumor-informed plasma or urinary DNA can occasionally persist after nephrectomy and track disease course, but RCC is a low-shedding malignancy and urine detection is inconsistent [20]. Postoperative proteins and EVs are additionally confounded by nephron loss and tubular injury.	Most persuasive when the alteration is matched to the index tumor, declines after treatment, and subsequently re-emerges with recurrence.	Nonviable molecular material, procedural release, uncertain clearance kinetics, poor shedding, and inability to localize the source.
**Field-level susceptibility**	Mutation or methylation positivity without a visible lesion	Morphologically normal urothelium may harbor cancer-associated mutations and clonal expansions, creating a recurrence-prone field that can release molecular material despite negative cystoscopy [12,13,45-47,144]. Broad methylation signals may similarly reflect occult tumor or altered urothelium.	UTUCs may arise through field cancerization or clonal seeding. Many paired UTUC and subsequent bladder tumors are clonally related, although independent primaries also occur [146].	Field-level interpretation is less established. Benign glandular expression, BPH, prostatitis, and sampling variability are more likely explanations for low-level urinary molecular positivity than a defined pan-prostatic field state [100,102,103].	Limited relevance as a defined cancer field state. Nonmalignant tubular injury and chronic kidney disease are more plausible sources of persistent renal epithelial, protein, RNA, or EV signals [8,147,148].	May identify a recurrence-prone or molecularly altered epithelial compartment without proving that a visible tumor is present.	Cannot reliably distinguish occult tumor, premalignant field, chronic injury, or the anatomical site of origin.
**Productive immune control**	Low tumor-specific signal with coordinated effector-cell or cytokine response	Most accessible during BCG therapy. A falling urinary tumor-DNA signal combined with coordinated local immune activation would be compatible with effective tumor control. Urine-derived immune cells and cytokines can be measured during BCG	Difficult to establish in voided urine because upper-tract immune products are diluted and mixed with bladder-derived cells and cytokines. Direct evidence is minimal.	Only indirectly accessible because prostate-derived material enters urine conditionally; prostatitis and benign inflammation substantially confound immune mediators.	Urinary immune signals may arise from RCC, nonmalignant renal parenchyma, systemic immune activation, infection, or treatment-associated nephritis; tumor-specific attribution is weak.	Falling tumor-informed signal together with a reproducible, treatment-associated effector pattern is more convincing than either component alone.	No validated urinary immune signature establishes productive antitumor immunity; cytokine magnitude alone is insufficient.

		treatment [37,52,72-74,152].					
**Engaged but insufficient immunity**	Persistent tumor signal despite measurable immune activation	Persistent urinary tumor DNA combined with BCG-induced immune activation may indicate that treatment has engaged the immune system without eliminating disease. High post-BCG urinary tumor DNA and tissue T-cell exhaustion have been associated with high-grade recurrence and BCG failure [145].	Biologically possible after topical or systemic treatment, but urine-based tumor–immune co-monitoring has not been validated.	Rarely measurable in urine because neither tumor burden nor intraprostatic immune activity is represented continuously.	Conceptually relevant during immunotherapy, but urinary inflammation cannot be separated readily from systemic immune activation or kidney toxicity.	Evidence of treatment engagement coexisting with molecular persistence suggests an active but inadequate response.	Immune activation may be nonspecific, treatment induced, or anatomically unrelated to the residual tumor.
**Immune failure or escape**	Persistent tumor signal with weak effector activity, T-cell exhaustion, or suppressive myeloid features	BCG failure has been associated with exhausted CD8-positive T-cell states and persistent urinary tumor DNA. BCG-treated urine also contains heterogeneous granulocytic and myeloid populations that may have stimulatory or suppressive functions [37,72,145].	Selective upper-tract immune sampling is uncommon, and evidence linking urinary immune phenotypes to treatment failure is lacking.	Tissue-level immune escape is biologically important, but urinary assays poorly represent the prostate tumor microenvironment.	RCC immune escape is central to systemic treatment resistance, but plasma and tissue are more informative than urine for monitoring this process.	Persistent tumor-informed material together with reproducible exhaustion or suppressive immune features would support inadequate immune control.	Most evidence pairs urinary tumor DNA with tissue immune phenotypes rather than demonstrating a fully validated urinary immune-escape signature.
**Nonspecific inflammation**	High inflammatory, leukocyte, protein, or EV signal with low or absent tumor-specific signal	BCG cystitis, urinary infection, recent TURBT, and instrumentation produce pyuria, cytokine release, granulocyte recruitment, and epithelial injury independent of viable tumor [27,153].	Ureteroscopy, stents, obstruction, topical therapy, and infection may generate hematuria and inflammation that are mixed with any tumor-derived signal in voided urine [91].	BPH, prostatitis, and urinary infection alter prostate-derived cells, RNA, proteins, and inflammatory mediators; histological inflammation can alter urinary PCA3 measurements [103].	Tubular injury, infection, CKD, systemic inflammation, and treatment nephrotoxicity alter urinary proteins, metabolites, and EV cargo independently of RCC [147,148].	Inflammation is present without convincing evidence of malignant burden.	May mimic tumor-associated immune activity or apparent treatment response, particularly when only nonspecific proteins or cytokines are measured.
**Treatment-selected and clonally evolving disease** [149-157]	Changing mutations, copy-number profiles, methylation, RNA expression, or EV cargo	Longitudinal urinary mutant-DNA profiling during neoadjuvant chemotherapy has demonstrated changing SNV and copy-number profiles compatible with	Clonal selection after ablation, chemotherapy, or immunotherapy is biologically plausible, but serial urine studies paired with repeat tumor tissue are scarce. Voided urine	Androgen-receptor adaptation and lineage plasticity are established mechanisms of progression, but urinary RNA provides only an incomplete and collection-	Plasma and tissue are generally more informative than urine. Personalized plasma and urine DNA profiling has occasionally tracked	Most convincing when serial molecular changes are clonally coherent and concordant with tissue, plasma,	Changing tumor burden, cell death, anatomical access, obstruction, hematuria, and treatment-induced

	during treatment	treatment-selected clonal evolution in individual patients [149]. More recent longitudinal studies show dynamic urinary tumor-DNA patterns, but prospective evidence that urine-guided evolutionary monitoring improves management remains limited [143].	also cannot reliably determine whether a new clone originates in the upper tract or bladder.	dependent representation of this evolution [150].	heterogeneity and disease course, but urinary detection remains inconsistent [20].	imaging, or clinical progression.	shedding can mimic clonal evolution.

Note: direct clinical evidence is strongest for bladder urinary tumor DNA, prostate pre-biopsy RNA classifiers, paired UTUC–bladder clonality, and selected bladder BCG studies. The proposed productive-immunity and immune-failure states are mechanistic frameworks rather than validated urinary classifications. UTUC residual-disease monitoring after kidney-sparing treatment and RCC post-nephrectomy urinary monitoring remain exploratory. **Abbreviations:** BCG, bacillus Calmette–Guérin; BPH, benign prostatic hyperplasia; CKD, chronic kidney disease; EV, extracellular vesicle; GG, Grade Group; PCA3, prostate cancer antigen 3; RCC, renal cell carcinoma; SNV, single-nucleotide variant; TURBT, transurethral resection of bladder tumor; UTUC, upper-tract urothelial carcinoma.

Suppl. Table S3. Framework for interpreting discordance between urinary biomarkers and conventional assessment.

Discordance domain	Observed pattern	Principal explanations	Illustrative clinical contexts	Interpretive implication and resolution strategy
**Analytical** [31,159-162,168-172]	Isolated weak positivity, an abrupt unexplained change, or fluctuation around the assay threshold	Contamination; analyte degradation; insufficient cellularity or cfDNA yield; amplification or sequencing error; batch effects; normalization failure; variable urine concentration; pre-analytical differences in collection, processing, storage, or specimen fraction	Applicable across cancers and assay classes, particularly low-abundance cfDNA, RNA, methylation, and EV assays	Establish reproducibility before invoking biological explanations. Review specimen identity, collection timing, hematuria, storage, assay quality metrics, and normalization; repeat testing under standardized conditions and, where possible, use an orthogonal assay.
**Anatomical** [11,17,20,75,144,146]	Urinary biomarker positive, but the presumed anatomical site is negative	Signal originates from another urinary-tract site; voided urine integrates both upper tracts and the bladder; prostate-derived material is collection dependent; RCC-associated material may arise from nonmalignant kidney tissue or systemic circulation	Positive voided urine with negative cystoscopy in bladder surveillance; positive urine after kidney-sparing UTUC treatment arising from the bladder or contralateral tract; RCC-associated protein or DNA without radiologically evident recurrence	Do not equate molecular detection with site localization. Reconstruct the anatomical route of entry and evaluate other plausible sources using cystoscopy, upper-tract imaging, selective urine or cytology when clinically indicated, collection-specific prostate assessment, and paired plasma analysis for RCC or systemic disease.
**Temporal: molecular lead time** [67,143]	Biomarker becomes positive before a lesion is visible endoscopically, radiologically, or histologically	Subclinical tumor burden below the resolution of the reference test; molecular recurrence preceding anatomical recurrence; intermittent visibility or sampling of a small lesion; expansion of a molecularly altered field	Urinary tumor DNA or methylation positive before cystoscopic bladder recurrence; tumor-associated urine signal with negative ureteroscopy; high-risk prostate RNA result despite negative MRI or biopsy	A positive result should not automatically be classified as false positive. Interpret the strength, tumor specificity, persistence, and trajectory of the signal and use it for risk refinement or selection of repeat conventional assessment; intervention requires a prospectively validated action threshold.
**Temporal: post-treatment persistence** [143,145,148,151,153]	Molecular, cellular, or inflammatory signal remains positive shortly after an intervention	Release of DNA or RNA from nonviable cells; surgical bleeding; epithelial injury; treatment-induced necrosis; delayed clearance; BCG-related inflammation; ureteroscopic or post-nephrectomy tissue injury	Post-TURBT tumor DNA; inflammatory markers after BCG; hematuria following ureteroscopy or ablation; proteins or EVs after nephrectomy	Interpret persistence against the expected kinetics of the analyte and intervention. A single early postoperative result is weaker evidence than failure to clear, sustained positivity, or re-emergence after an initial decline. Serial sampling should use treatment-specific and assay-specific time points.
**Biological: field alteration or multicentricity** [12,13,45-47,146]	Mutation or methylation signal detected without a visible lesion	Clonally altered but morphologically normal urothelium; carcinogen-associated field cancerization; intraluminal seeding; occult CIS; clonally related disease at another urothelial site	Mutation-positive or methylation-positive urine with negative cystoscopy; molecular detection after removal of the index bladder tumor; recurrent bladder signal following treatment of UTUC	Treat the result as evidence of a molecularly abnormal or recurrence-prone urothelial compartment, not proof of a localized tumor. Serial persistence, tumor matching, enhanced cystoscopic assessment, and upper-tract evaluation can help distinguish occult disease from nonlocalizing field susceptibility.

**Biological: inflammation or tissue injury** [8,27,100,103,147,148,153]	Immune-cell, cytokine, protein, metabolite, or EV signal positive while tumor-specific markers are absent or low	Infection; BCG cystitis; recent instrumentation; prostatitis or BPH; obstruction; tubular injury; CKD; systemic inflammation; treatment-associated nephritis	High urinary cytokines after BCG without persistent tumor DNA; prostate RNA or protein changes during prostatitis; RCC-associated proteins or EVs in the presence of renal injury	Establish whether the assay measures malignancy, host response, or both. Evaluate infection, inflammation, hematuria, renal function, recent procedures, and treatment exposure. Repeat measurement after resolution of reversible inflammatory or injury states when clinically appropriate.
**Biological: poor or heterogeneous shedding** [20,25,75,101,144]	Biomarker negative despite tumor detected by endoscopy, imaging, or biopsy	Small or low-grade tumor; low tumor fraction; intermittent shedding; luminal obstruction; anatomical separation from urine; inadequate prostate enrichment; low ctDNA release by RCC; loss or absence of the assay target	Low-grade bladder cancer; small or obstructed UTUC; MRI-visible or biopsy-proven prostate cancer with a low-risk urinary score; localized RCC with undetectable urinary cfDNA	A negative urinary assay cannot overrule a positive reference test. Assess whether the specimen and assay were capable of representing that tumor, considering anatomical access, collection method, tumor subtype, specimen fraction, and target coverage. Tissue profiling or an alternative biofluid may identify target mismatch or non-shedding disease.
**Reference-standard** [91,101,105,106,110-114]	Biomarker positive but cystoscopy, ureteroscopy, MRI, imaging, or biopsy is negative	Flat or visually subtle CIS; small UTUC lesion; inadequate ureteroscopic biopsy; MRI-invisible prostate cancer; spatial undersampling by prostate biopsy; radiologically occult or subthreshold disease	Positive bladder biomarker with normal white-light cystoscopy; high-risk urinary prostate score with negative or equivocal MRI; selective urine positivity with nondiagnostic ureteroscopic biopsy	Recognize that conventional tests are imperfect comparators rather than absolute truth standards. Reassess pretest risk and biomarker specificity and consider enhanced imaging, repeat or targeted biopsy, enhanced cystoscopy, selective sampling, or longitudinal observation rather than automatically relabeling the biomarker as false positive.
**Treatment-state** [27,141,142,145,148,151-153]	Direction or magnitude of the urinary signal changes during therapy without concordant clinical change	BCG-induced inflammation; chemotherapy-mediated cell death and transient DNA release; treatment-selected clonal expansion; altered prostatic secretion during androgen-pathway therapy; VEGF-inhibitor or immune-checkpoint-inhibitor nephrotoxicity; treatment-related changes in filtration or shedding	Rising urinary DNA early during cytotoxic treatment; persistent cytokine elevation during BCG; altered urinary renal injury markers during systemic RCC therapy	Treatment-naive thresholds should not be transferred uncritically to on-treatment samples. Interpret changes relative to a pretreatment baseline and alongside imaging, tumor-informed molecular measurements, clinical response, urinalysis, renal function, and—where relevant—paired plasma.

Abbreviations: BCG, bacillus Calmette–Guérin; BPH, benign prostatic hyperplasia; cfDNA, cell-free DNA; CIS, carcinoma in situ; CKD, chronic kidney disease; EV, extracellular vesicle; MRI, magnetic resonance imaging; RCC, renal cell carcinoma; TURBT, transurethral resection of bladder tumor; UTUC, upper-tract urothelial carcinoma.

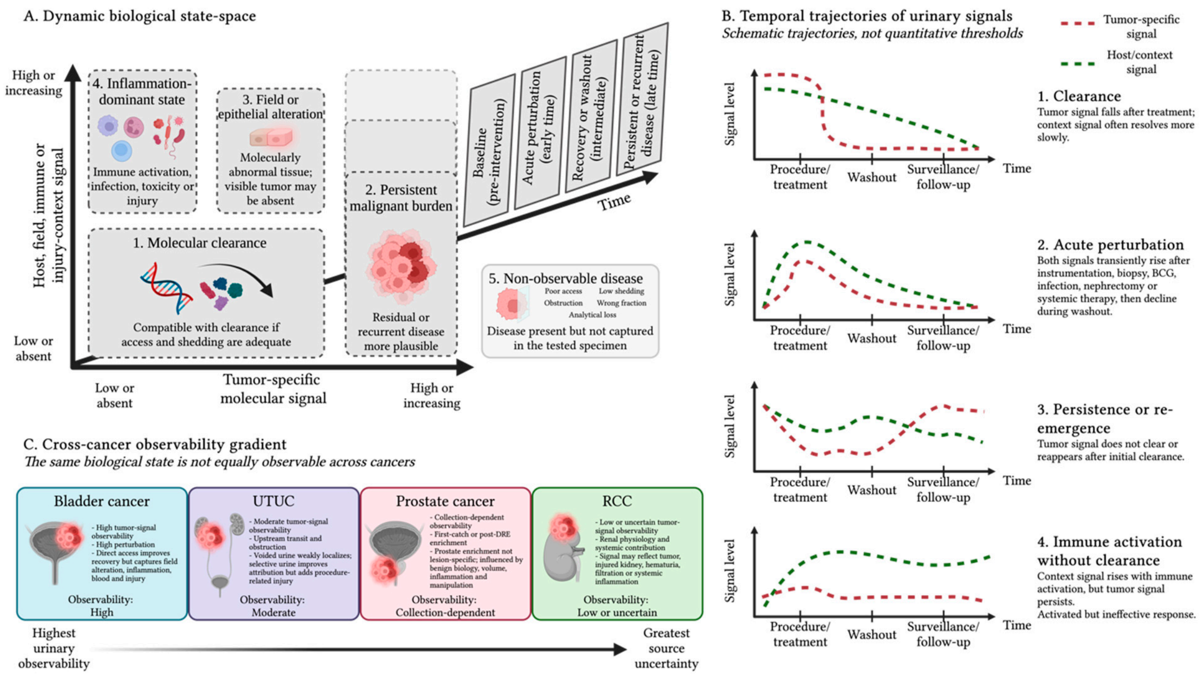

Suppl. Figure S1. Biological state, temporal trajectory and urinary observability in longitudinal biomarker interpretation.

A. Urinary biomarker results reflect dynamic biological states created by the interaction between tumor-specific molecular signal and host, field, immune or injury-context signal. A positive result may indicate persistent malignant burden, field alteration, inflammation-dominant biology or transient perturbation, whereas a negative result supports clearance only when access, shedding and analytical recovery are adequate. B. Schematic temporal trajectories show how tumor-specific and host/context signals may behave after procedure or treatment. Acute perturbation can transiently increase urinary signal, whereas post-washout persistence or re-emergence is more compatible with residual or recurrent disease. Immune activation without molecular clearance may indicate treatment engagement without tumor eradication. C. Urinary observability differs across cancers. Bladder cancer has the highest access; UTUC is limited by upstream dilution, obstruction and localization uncertainty; prostate cancer is collection-dependent; and RCC has the greatest source-attribution uncertainty. Curves are conceptual and do not represent quantitative thresholds. **Abbreviations**: BCG, bacillus Calmette–Guérin; DRE, digital rectal examination; RCC, renal cell carcinoma; UTUC, upper-tract urothelial carcinoma. Created in BioRender. Asanova, A. (2026) https://BioRender.com/vzyouqs (Accessed 17 June 2026).
